# STAT3 activation by E6 is essential for the differentiation-dependent HPV18 life cycle

**DOI:** 10.1371/journal.ppat.1006975

**Published:** 2018-04-09

**Authors:** Ethan L. Morgan, Christopher W. Wasson, Lucy Hanson, David Kealy, Ieisha Pentland, Victoria McGuire, Cinzia Scarpini, Nicholas Coleman, J. Simon C. Arthur, Joanna L. Parish, Sally Roberts, Andrew Macdonald

**Affiliations:** 1 School of Molecular and Cellular Biology, Faculty of Biological Sciences and Astbury Centre for Structural Molecular Biology, University of Leeds, Leeds, United Kingdom; 2 Institute of Cancer and Genomic Sciences, College of Medical and Dental Sciences, University of Birmingham, Edgbaston, Birmingham, United Kingdom; 3 Division of Cell Signalling and Immunology, College of Life Sciences, Sir James Black Centre, University of Dundee, Dundee, United Kingdom; 4 Department of Pathology, University of Cambridge, Cambridge, United Kingdom; Fred Hutchinson Cancer Research Center, UNITED STATES

## Abstract

Human papillomaviruses (HPV) activate a number of host factors to control their differentiation-dependent life cycles. The transcription factor signal transducer and activator of transcription (STAT)-3 is important for cell cycle progression and cell survival in response to cytokines and growth factors. STAT3 requires phosphorylation on Ser727, in addition to phosphorylation on Tyr705 to be transcriptionally active. In this study, we show that STAT3 is essential for the HPV life cycle in undifferentiated and differentiated keratinocytes. Primary human keratinocytes containing high-risk HPV18 genomes display enhanced STAT3 phosphorylation compared to normal keratinocytes. Expression of the E6 oncoprotein is sufficient to induce the dual phosphorylation of STAT3 at Ser727 and Tyr705 by a mechanism requiring Janus kinases and members of the MAPK family. E6-mediated activation of STAT3 induces the transcription of STAT3 responsive genes including cyclin D1 and Bcl-xL. Silencing of STAT3 protein expression by siRNA or inhibition of STAT3 activation by small molecule inhibitors, or by expression of dominant negative STAT3 phosphorylation site mutants, results in blockade of cell cycle progression. Loss of active STAT3 impairs HPV gene expression and prevents episome maintenance in undifferentiated keratinocytes and upon differentiation, lack of active STAT3 abolishes virus genome amplification and late gene expression. Organotypic raft cultures of HPV18 containing keratinocytes expressing a phosphorylation site STAT3 mutant display a profound reduction in suprabasal hyperplasia, which correlates with a loss of cyclin B1 expression and increased differentiation. Finally, increased STAT3 expression and phosphorylation is observed in HPV positive cervical disease biopsies compared to control samples, highlighting a role for STAT3 activation in cervical carcinogenesis. In summary, our data provides evidence of a critical role for STAT3 in the HPV18 life cycle.

## Author summary

Human papillomaviruses (HPV) are the leading cause of viral induced cancers worldwide. HPV are the causative agents of cervical cancers and an increasing number of head and neck cancers. HPV infections are dependant on the manipulation of the host cell for their replication and this may result in diseases such as cancer. The STAT3 transcription factor, a known driver of cancer progression, is often over active in HPV-associated cancers; however, its role in the life cycle of HPV has not been studied. Using primary cell culture models we provide the first evidence demonstrating that HPV increases both the phosphorylation and activity of STAT3 and that this is required for viral gene expression and replication. Importantly, inhibition of STAT3 by small molecule inhibitors or expression of STAT3 mutants that cannot be phosphorylated impairs the HPV life cycle. Finally, we demonstrate that STAT3 phosphorylation is increased during cervical disease progression, highlighting the potential of STAT3 as a novel therapeutic target in HPV-associated cancers.

## Introduction

Human papillomaviruses (HPVs) are small, non-enveloped double-stranded DNA viruses that show a tropism for squamous epithelial cells of the skin epidermis, oral and ano-genital mucosa. Infection with HPV is associated with a spectrum of clinical manifestations ranging from common warts to cancers [1]. Whilst >200 types of HPV have been identified (https://pave.niaid.nih.gov/), only a sub-set of these are classed as high-risk due to their association with malignancy. High-risk HPVs are responsible for >99% of cervical cancer cases and a growing number of oropharyngeal carcinomas [2,3]. In particular, high-risk HPV16 and HPV18 are detected in >70% of cervical cancer cases and over 90% of other HPV positive cancers [4].

The HPV life cycle is intrinsically linked to the terminal differentiation programme of the epithelial tissues they infect, with productive replication restricted to differentiated suprabasal cells. Following infection of mitotically active cells within the basal layer of the epithelium, HPV genomes are established as low copy (~100 copies) number episomes [5]. Upon differentiation, HPV infected cells remain active in the cell cycle and re-enter S/G2 phases for virus genome amplification. In the upper layers of the epithelium, infected cells exit the cell cycle and complete differentiation, enabling transit to the late stage of infection, where the late promoter is activated to drive late gene expression prior to virion production [5]. HPV replication is dependent on host factors, which are mainly controlled by the activities of the virus encoded E5, E6 and E7 proteins. Whilst the role of the E5 protein is less understood [6], the E6 and E7 oncoproteins are pivotal in the productive life cycle as well as in the development of anogenital cancers [7]. E7 proteins promote S phase re-entry in the differentiated strata via an ability to bind and inactivate the pocket family proteins pRb, p107 and p130. These interactions result in release of the transcription factor E2F, causing cell cycle progression in cells that would normally be undergoing differentiation [8,9]. E6 recruits the cellular ubiquitin ligase E6-associated protein (E6AP) into a protein complex with the tumour suppressor protein p53, resulting in its degradation [10,11]. In addition, high-risk E6 proteins bind and degrade a select group of PSD95/DLG/ZO-1 (PDZ) domain containing proteins [12]. Disruption of either of these functions interferes with the HPV life cycle [13–16]. Despite our increased understanding of the HPV life cycle, there is still a clear need to understand how HPV and the host cell proteins it manipulates determine the outcome of HPV infection to define novel strategies to treat HPV infections.

STAT3 is a member of the signal transducer and activator (STAT) family of transcription factors that were originally discovered through their capacity to mediate transcription in response to interferons [17]. Binding of cytokines and growth factors to cell surface receptors results in activation of STAT3, which typically involves phosphorylation of a tyrosine (Y705) residue [18]. Phosphorylation is primarily mediated by receptor-associated kinases (e.g. Janus family kinases (JAK)) and receptor tyrosine kinases (e.g. epidermal growth factor receptor (EGFR)), but can also be accomplished by non-receptor tyrosine kinases such as Src [19,20]. Tyrosine phosphorylated STAT3 molecules form activated dimers and translocate to the nucleus where they initiate a programme of gene expression controlling fundamental biological processes such as proliferation, apoptosis, immune regulation and differentiation [21]. STAT3 is also subject to stimulus-dependent phosphorylation of a serine (S727) residue in its transactivation domain [18]. The function of this additional phosphorylation event remains controversial, as the modification has been reported to enhance and suppress STAT3 transcriptional activity [22,23].

In stratified epithelia, STAT3 drives proliferation and is required to maintain cells in an undifferentiated state by actively impairing differentiation [24–26]. Genetically engineered STAT3 knockout mice show reduced epithelial proliferation and suffer a compromised wound healing response [27]. In addition, both maintenance and renewal of keratinocyte stem cells is impaired. In contrast, mice engineered to express a constitutively active STAT3 in the proliferative compartment of the epithelium exhibit a disturbed gene expression profile resulting in an enrichment of expression of genes associated with epithelial to mesenchymal transition [28]. Keratinocytes from these mice display an increased survival rate in response to chemical insult or exposure to UV radiation [29]. Constitutive activation of STAT3 is also a feature of many human malignancies including cervical, and head and neck cancers, and is associated with a poor prognosis in various tumours [30]. Such aberrant activation of STAT3 contributes to tumour initiation, progression, immune evasion and metastasis [21,31]. Despite a clear link between STAT3 activation and HPV-associated cancers, it is not known whether STAT3 contributes to the productive HPV life cycle. This is surprising given the important role of STAT3 in maintaining an undifferentiated phenotype in keratinocytes.

Here, we show that STAT3 phosphorylation is increased in HPV18-positive cells, and maintained during differentiation, through the activities of JAK and MAPK family kinases. Increased STAT3 activity is associated with host gene expression changes, including over-expression of proteins required for cell cycle progression and cell survival. Importantly, blockade of STAT3 activity through small molecule inhibitors, siRNA mediated depletion or over-expression of dominant negative phosphorylation null STAT3 mutants impairs viral gene expression and viral DNA replication in undifferentiated and differentiated cells. Finally, we demonstrate that phosphorylation is essential for STAT3 function in the HPV replication cycle and is frequently increased in HPV-positive cervical disease. Thus, STAT3 is a critical host factor required during the high-risk HPV life cycle.

## Results

### HPV18 enhances STAT3 phosphorylation in primary human keratinocytes

**Fig 1:**
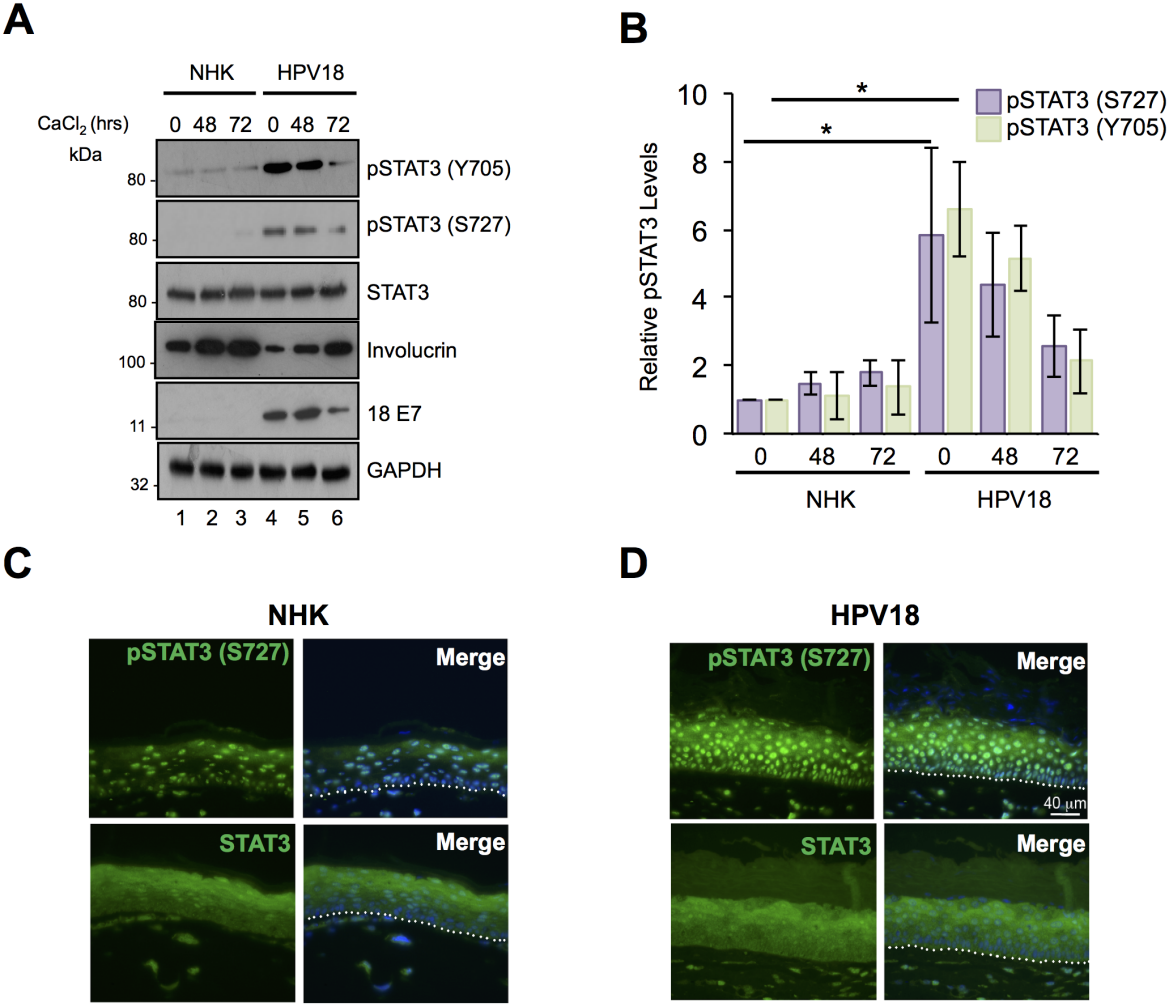
HPV18 induces the activation of STAT3 in primary human keratinocytes. **A)** Representative western blots of normal human keratinocytes (NHK) and HPV18-containing keratinocytes subjected to high calcium differentiation and analysed for STAT3 phosphorylation using specific antibodies against pS727 STAT3, pY705 STAT3 and total STAT3. Involucrin expression is a marker of differentiation and E7 demonstrates the presence of HPV18 in the samples. GAPDH serves as a loading control. **B)** Quantification of the protein band intensities in A) standardised to GAPDH levels. Bars represent the means ± standard deviation from 4 independent biological repeats using 2 donor cell lines. *P<0.05 (Student’s t-test). Representative sections of organotypic raft cultures from **C)** NHK and **D)** HPV18-containing keratinocytes stained with antibodies specific for pS727 STAT3 and total STAT3 (green) and counterstained with DAPI to highlight the nuclei (blue in the merged panels). Images were acquired using identical exposure times. Scale bar, 40 μm. White dotted lines indicate the basal cell layer.

To investigate the role of STAT3 in the HPV18 life cycle, stable cell lines harbouring HPV18 episomes were generated from two primary foreskin keratinocyte donors. To exclude donor effects, all experiments were performed using both donor lines and representative data are presented. Levels of STAT3 phosphorylation were measured in normal human keratinocytes (NHK) and HPV18-containing cells by western blotting. In undifferentiated cells, phosphorylation of the major tyrosine (Y705) and serine (S727) residues was enhanced significantly in keratinocytes harbouring HPV18 compared to NHK donor controls (Fig 1A, compare lanes 1 and 4, Fig 1B, showing combined data from two donors; N = 8 p<0.05). To ascertain whether STAT3 phosphorylation was further modulated during keratinocyte differentiation, monolayer cultures of NHK and HPV18-containing cells were cultured in high calcium media for 72 hours and samples taken for western blot analysis. Keratinocyte differentiation was confirmed by increased involucrin expression (Fig 1A). Importantly, whilst subject to a decline, enhanced STAT3 phosphorylation was maintained at detectable levels in the HPV18-containing cells during differentiation (Fig 1A, compare lanes 2 and 5, 3 and 6). In contrast, HPV18 had no effect upon the total levels of STAT3 protein in the primary keratinocytes (Fig 1A). Comparable STAT3 phosphorylation changes were observed in both donor samples.

Next, we confirmed our findings in an additional model of keratinocyte differentiation. NHK and HPV18-containing cells were stratified in organotypic raft culture for 14 days; this method recapitulates all stages of the HPV life cycle [32]. Raft sections were stained with antibodies detecting the total and phosphorylated forms of STAT3 (representative sections from one donor shown in Fig 1C and 1D). Staining of total STAT3 protein was comparable between the donor-matched NHK and HPV18-containing rafts. S727 phosphorylated STAT3 was evident in both the basal and suprabasal layers of the NHK and HPV18 containing rafts. However, the level of the S727 phosphorylated form of STAT3 was elevated in the presence of HPV18 (Fig 1C and 1D). We were not able to successfully stain raft cultures with the Y705 STAT3 phosphorylation specific antibody and so could not confirm the results obtained from our calcium differentiation assays. However, our data clearly demonstrate that primary keratinocytes harbouring HPV18 genomes exhibit increased STAT3 phosphorylation.

### HPV18 E6 is necessary and sufficient for the dual phosphorylation and activation of STAT3

**Fig 2:**
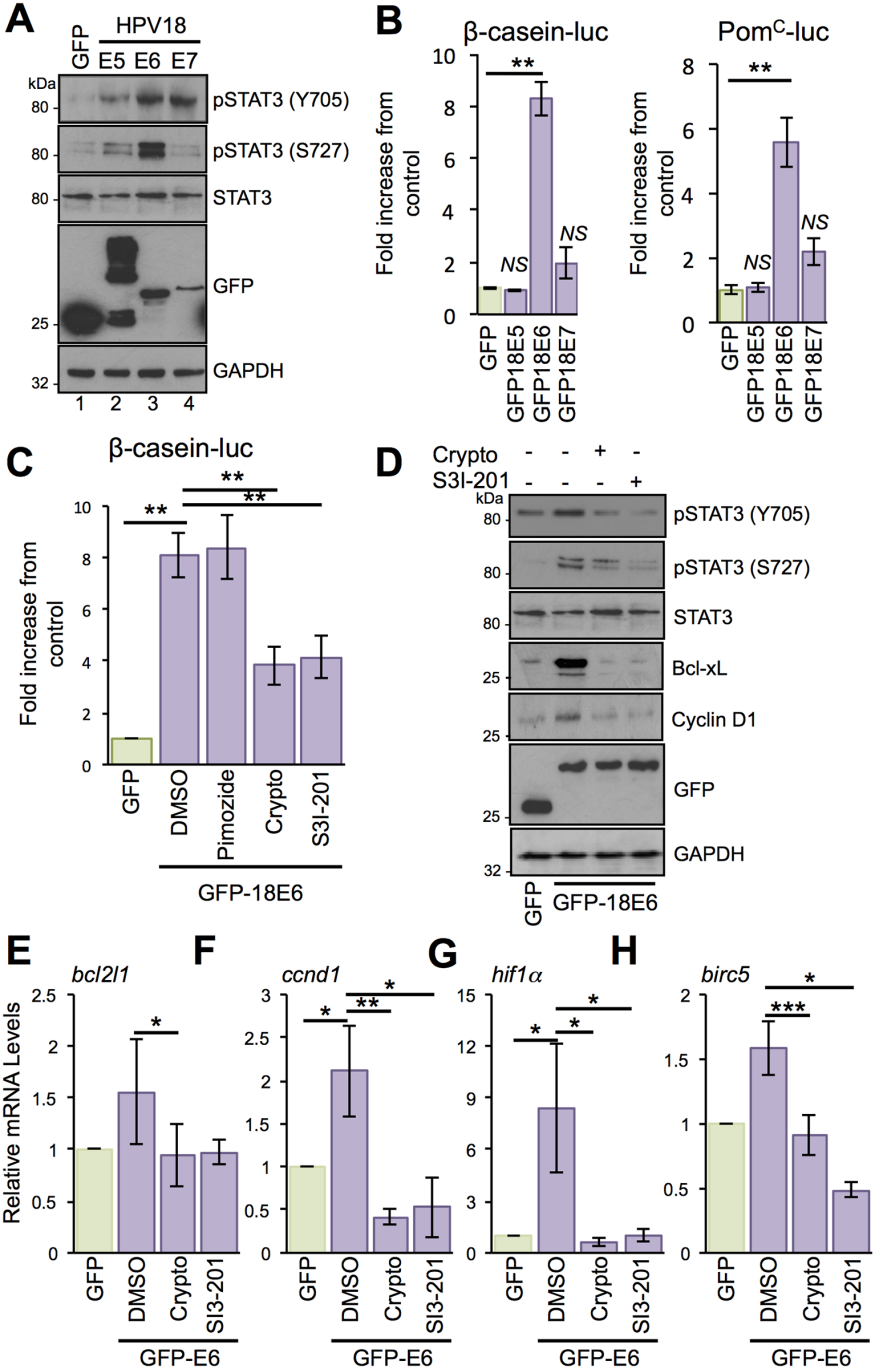
HPV E6 promotes the dual phosphorylation and activation of STAT3. **A)** Representative western blot of C33a cells transfected with GFP tagged HPV18 oncoproteins E5, E6 or E7 and analysed for STAT3 activation using specific antibodies detecting phosphorylated and total STAT3. Expression of HPV oncoproteins was confirmed using a GFP antibody. GAPDH served as a loading control. **B)** Representative luciferase reporter assays from C33a cells co-transfected with GFP tagged HPV18 oncoproteins E5, E6 or E7 and either a β-casein promoter reporter plasmid or a Pom^C^ reporter plasmid, which contain STAT3 binding sites, and promoter activity measured using a dual-luciferase system. Data are presented as relative to the GFP transfected control cells. Bars represent the means ± standard deviation from at least three independent biological repeats. **P<0.01, *N*.*S*. = not significant (Student’s t-test). **C)** Representative luciferase reporter assays from C33a cells co-transfected with GFP tagged E6 and a β-casein promoter reporter plasmid treated with the STAT3 inhibitors cryptotanshinone and S3I-201, and the STAT5 inhibitor pimozide. Promoter activity was measured using a dual-luciferase system. Data are presented as relative to the GFP transfected control. Bars represent the means ± standard deviation from at least three independent biological repeats. **P<0.01 (Student’s t-test). **D)** Representative western blot of C33a cells transfected with GFP or GFP tagged HPV18 E6, untreated or treated with the STAT3 inhibitors as above and analysed for total and phosphorylated STAT3 (Y705 and S727), the expression of cyclin D1 and Bcl-xL and GFP to demonstrate expression of the GFP-18E6 fusion protein. GAPDH expression was used as a loading control. Data shown are representative of at least three biological repeats. **E-H)** C33a cells were transiently transfected with GFP or GFP tagged HPV18 E6 and left untreated or treated with the STAT3 inhibitors as above and RNA was extracted for qRT-PCR analysis of the indicated STAT3 dependent genes. Samples were normalized against U6 mRNA levels. Representative data are presented relative to the GFP transfected control. Bars are the means ± standard deviation from at least three biological repeats. *P<0.05, **P<0.01, ***P<0.001 (Student’s t-test).

Next, we hypothesised that a virus-encoded oncoprotein (E5, E6 and E7) promotes the increased STAT3 phosphorylation observed in the HPV18 life cycle model. To determine whether one or more of the HPV18 oncoproteins was responsible for STAT3 activation, the levels of STAT3 phosphorylation were measured by western blot analysis of C33a (an HPV-negative cervical carcinoma cell line) cells expressing individual GFP-tagged HPV18 oncoproteins (Fig 2A). Interestingly, all three oncoproteins increased Y705 STAT3 phosphorylation compared to cells expressing GFP alone (compare lanes 1 with 2, 3 and 4); E5 by average of 3 fold (p<0.05), E6 by an average of 4.1 fold (p<0.05) and E7 by an average of 3.6 fold (p<0.05) (Fig 2A, lanes 2–4). Overexpression of both E5 and E7 led to a small increase in S727 STAT3 phosphorylation, however, E6 expression increased S727 phosphorylation significantly by an average of 5.6 fold (p<0.01) (Fig 2A, lane 3). In agreement with our observations from primary keratinocytes, the presence of the HPV oncoproteins did not alter the level of total STAT3 protein, which remained similar to the GFP control.

Next, we investigated whether the viral oncoproteins are responsible for the increased STAT3 phosphorylation observed in primary keratinocytes harbouring the HPV18 genomes. For this, we silenced E6 expression in the HPV18 life cycle model using a pool of E6-specific siRNA and compared the levels of phosphorylated STAT3 to those of cells transfected with a scrambled control siRNA (S1A Fig). Treatment with the E6-siRNA resulted in a loss of E6 expression coupled to an increase in levels of the E6 target p53 (S1A Fig). Importantly, the loss of E6 also correlated with a significant reduction in both Y705 and S727 STAT3 phosphorylation (p<0.001). Our E6 depletion approach also resulted in decreased E7 expression (average of 55%) (S1A Fig). To rule out any contributory role for this oncoprotein, we tested whether targeted siRNA-mediated depletion of E7 also affected STAT3 phosphorylation. Treatment of HPV18 genome-containing cells with E7-specific siRNA reduced E7 expression by approximately 60% without impacting on either E6 expression or STAT3 phosphorylation (S1B Fig). To test the contribution of the E5 oncoprotein we utilised our recently generated primary keratinocyte lines containing an E5 knockout HPV18 genome [33]. Using this system we showed that loss of E5 did not reduce STAT3 phosphorylation in either monolayer cultures differentiated in high calcium media or organotypic raft cultures (S1C and S1D Fig). In summary, these data indicate that E6 is the predominant protein responsible for increasing the STAT3 phosphorylation observed in HPV18-containing keratinocytes.

To address whether increased STAT3 phosphorylation correlated with enhanced STAT3 transactivation, we measured the promoter activity of two STAT3-dependent reporter plasmids. C33a cells were co-transfected with isolated HPV oncoproteins and reporter plasmids driving firefly luciferase from the β-casein [34] and pro-opiomelanocortin (Pom^C^) [35] promoters. Expression of HPV18 E5 and E7 did not significantly increase STAT3-dependent luciferase expression. Conversely, expression of HPV18 E6 led to an average 8-fold increase in β-casein promoter-driven luciferase (p<0.01) and a 5.5-fold increase in Pom^C^-driven luciferase (p<0.01) (Fig 2B). Whilst these reporter constructs have been widely used to monitor the activation of STAT3, they can also be responsive to other members of the STAT family of transcription factors [35,36]. In this regard, a recent study has shown that HPV activates STAT5, which is necessary for HPV31 genome amplification [37]. To exclude the possibility that the observed increase in luciferase expression was a result of STAT5 activation, we used a pharmacological approach to specifically block the activation of STAT3 and STAT5 (Fig 2C). Transfected C33a cells were treated with the STAT5 inhibitor pimozide [37] or two chemically distinct STAT3 inhibitors, cryptotanshinone and S3I-201. Firstly, we confirmed that cryptotanshinone (S2A Fig) treatment did not affect STAT5 phosphorylation in HPV18-containing keratinocytes. Importantly, cells treated with pimozide showed no reduction in luciferase expression whereas in contrast, treatment with the two STAT3 inhibitors resulted in a significant (p<0.01) reduction in β-casein-driven luciferase expression (Fig 2C).

We next assessed if E6 could induce the expression of endogenous STAT3-dependent gene products. Expression of HPV18 E6 in C33a cells led to increased expression of cyclin D1 and Bcl-_X_L, two characterised STAT3-dependent gene products (Fig 2D). To confirm that the induction of cyclin D1 and Bcl-xL was STAT3-dependent, E6 expressing cells were treated with cryptotanshinone and S3I-201. Treatment with either inhibitor reduced STAT3 phosphorylation at both tyrosine and serine residues, but had minimal impact on levels of total STAT3 protein. In addition, inhibitor treatment reduced cyclin D1 and Bcl-xL expression to control levels (Fig 2D). Quantitative reverse transcriptase-PCR (qRT-PCR) revealed E6-dependent increases in cyclin D1(*ccnd1*) and Bcl-_X_L (*bcl2l1*) mRNA transcripts, and these increases were sensitive to treatment with STAT3 inhibitors (*ccnd1* plus crypto p<0.01 and *bcl2l1* plus crypto p<0.05) (Fig 2E and 2F). Additional STAT3-dependent genes, including HIF1α and Survivin (*birc5*), were up-regulated by E6 in a STAT3-dependent manner (*hif1*α plus crypto p<0.05 and *birc5* plus crypto p<0.001) (Fig 2G and 2H). Based on these results, we conclude that the E6 oncoprotein increases the transcription of STAT3-dependent genes.

### STAT3 is phosphorylated in cells expressing E6 mutants defective for E6AP binding, p53 degradation and PDZ domain-binding

**Fig 3:**
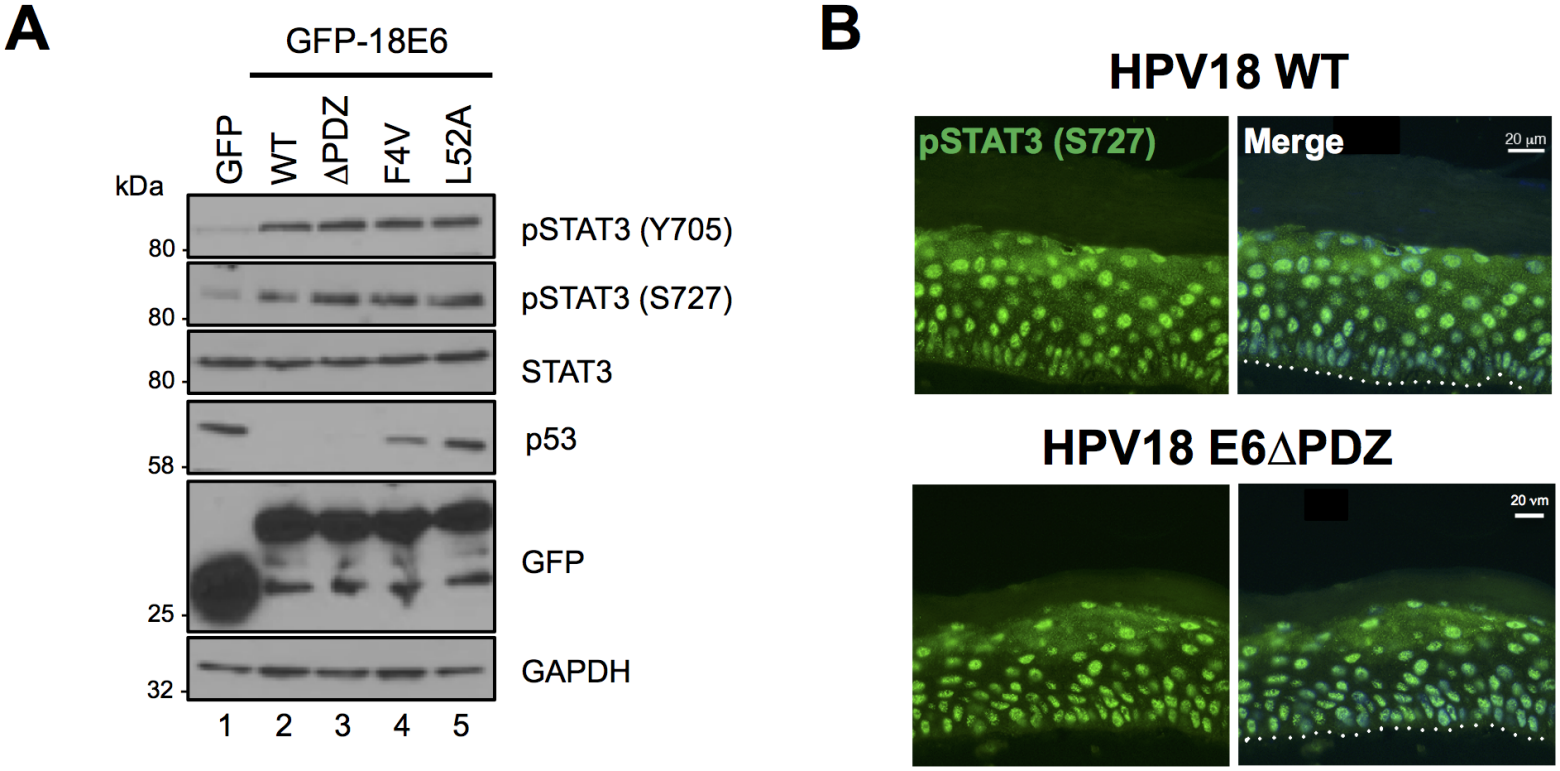
STAT3 is phosphorylated in cells expressing E6 defective for E6AP binding, p53 degradation and PDZ domain binding. **A)** Representative western blot of C33a cells transfected with GFP or GFP tagged HPV18 E6 wild type, E6 ΔPDZ, E6 F4V or E6 L52A and analysed using antibodies detecting total and phosphorylated STAT3 and p53. GAPDH expression was used as a loading control and GFP confirmed expression of the E6 proteins. Data presented are representative of at least three independent experiments. **B)** Organotypic raft sections were stained with an antibody specifically detecting STAT3 pS727 (green) and counterstained with DAPI to highlight the nuclei (blue—in merged panels). Images were acquired with identical exposure times. The dotted line indicates the basal cell layer. Scale bar, 20 μm.

Whilst E6 proteins lack intrinsic enzymatic activities, those E6 proteins encoded by high-risk HPVs are able to interact with key cellular partners including E6AP, p53 and a number of PDZ-domain containing proteins to modulate cellular functions [38–40]. Therefore, we tested whether these interactions were required for the E6-mediated increase in STAT3 phosphorylation observed in HPV-positive cells. To this end, STAT3 phosphorylation was measured in cells expressing wild type and mutant HPV18 E6 proteins deficient in their ability to bind p53, E6AP or PDZ domains. Valine substitution of phenylalanine at amino acid position four (F4V) generates an E6 protein incapable of destabilizing p53 and in a HPV18 life cycle model is deficient in supporting viral DNA amplification [41]. Whilst deficient for inhibiting p53 destabilization, the E6 F4V mutant enhanced STAT3 phosphorylation to levels comparable with wild type E6 (Fig 3A; compare lanes 2 and 4). E6 expression leads to the degradation of p53 by virtue of its interaction with the E6AP E3 ubiquitin ligase. Expression of an E6 mutant unable to interact with E6AP (L52A) [42] impaired p53 degradation, but retained the ability to enhance STAT3 phosphorylation (Fig 3A; compare lanes 2 and 5). To test whether E6 interaction with PDZ proteins was required for increased STAT3 phosphorylation, we engineered HPV18 E6 ΔPDZ, which lacks the C-terminal four amino acid PDZ-binding motif and cannot bind to PDZ domains [13]. When expressed in C33a cells, this mutant protein induced STAT3 phosphorylation to wild type E6 levels (Fig 3A; compare lanes 2 and 3). Finally, we confirmed that the PDZ-binding domain of E6 was not required for STAT3 phosphorylation in the context of the entire HPV genome in a differentiating epithelium. Organotypic raft cultures were generated from NHK harbouring wild type and ΔPDZ HPV18 genomes [13]. Rafts were stained with an antibody that detects the pS727 form of STAT3. A similar pattern of STAT3 S727 phosphorylation was observed throughout the basal and suprabasal layers of the HPV18 wild type and ΔPDZ containing rafts (Fig 3B). Together these data demonstrate that STAT3 phosphorylation is increased in cells expressing HPV18 by a mechanism independent of the p53 destabilizing and PDZ binding functions of E6.

### Janus kinases (JAK) are responsible for STAT3 Y705 phosphorylation in HPV18 containing keratinocytes

**Fig 4:**
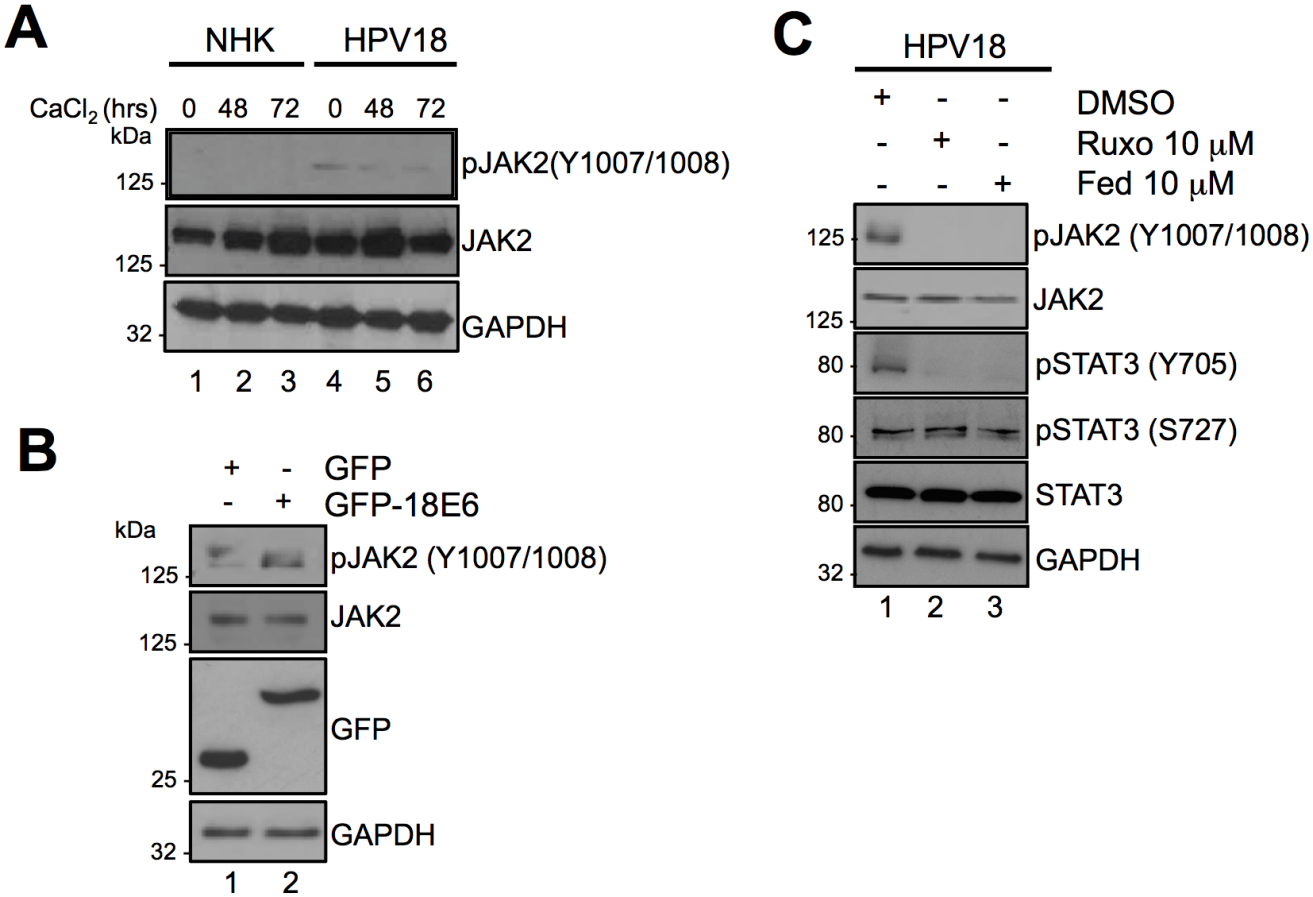
Janus kinases (JAK) are responsible for mediating Y705 phosphorylation in E6 and HPV18 containing keratinocytes. **A)** Representative western blot of keratinocytes subjected to high calcium differentiation analysed with antibodies specific for the total and phosphorylated forms of JAK2 (Y1007/1008). GAPDH served as a loading control. Data shown are representative of at least three independent biological repeats. **B)** Representative western blot of C33a cells transfected with GFP and GFP tagged HPV18 E6 and analysed using specific antibodies detecting phosphorylated (Y1007/1008) and total JAK2. Expression of E6 was confirmed using a GFP antibody. GAPDH served as a loading control. **C)** Representative western blots of HPV18 containing keratinocytes incubated with the JAK1/2 inhibitor Ruxolitinib or JAK2 inhibitor Fedratinib analyzed with antibodies detecting total and phosphorylated forms of JAK2 and STAT3. GAPDH served as a loading control. Data shown are representative of at least three independent biological repeats.

Janus family receptor-associated tyrosine kinases are the most common kinases responsible for mediating STAT3 Y705 phosphorylation, which is deemed necessary for STAT3 activation [30]. Previous studies have shown that treatment of SiHa HPV16-positive cervical cancer cells with AG490, a non-specific JAK2 inhibitor, prevented STAT3 Y705 phosphorylation [43], however, such studies have not been performed in primary keratinocytes. To address this, we first confirmed that JAK were active in primary keratinocytes harbouring HPV18 by western blot analysis for tyrosine phosphorylated JAK2. Levels of phosphorylated JAK2 were higher in HPV18-containing keratinocytes compared to NHK controls, and were retained during calcium-mediated differentiation (Fig 4A). Next, we showed that increased JAK phosphorylation was E6-dependent by overexpressing a GFP HPV18 E6 fusion protein in C33a cells. Western blot analysis showed an increase in JAK phosphorylation in C33a cells expressing GFP-18E6 compared to GFP control (Fig 4B; compare lanes 1 and 2). Lastly, we incubated HPV18-containing keratinocytes with the highly specific clinically available JAK1/2 inhibitor Ruxolitinib [44], or the JAK2 inhibitor Fedratinib [45]. Treatment with either inhibitor led to a marked reduction in STAT3 Y705 phosphorylation without affecting S727 phosphorylation (Fig 4C; compare lane 1 to 2 and 3). These results indicate that JAK2 mediates the STAT3 Y705 phosphorylation in HPV18-containing primary keratinocytes.

### Functionally redundant MAPK proteins mediate STAT3 S727 phosphorylation in HPV18-containing keratinocytes

**Fig 5:**
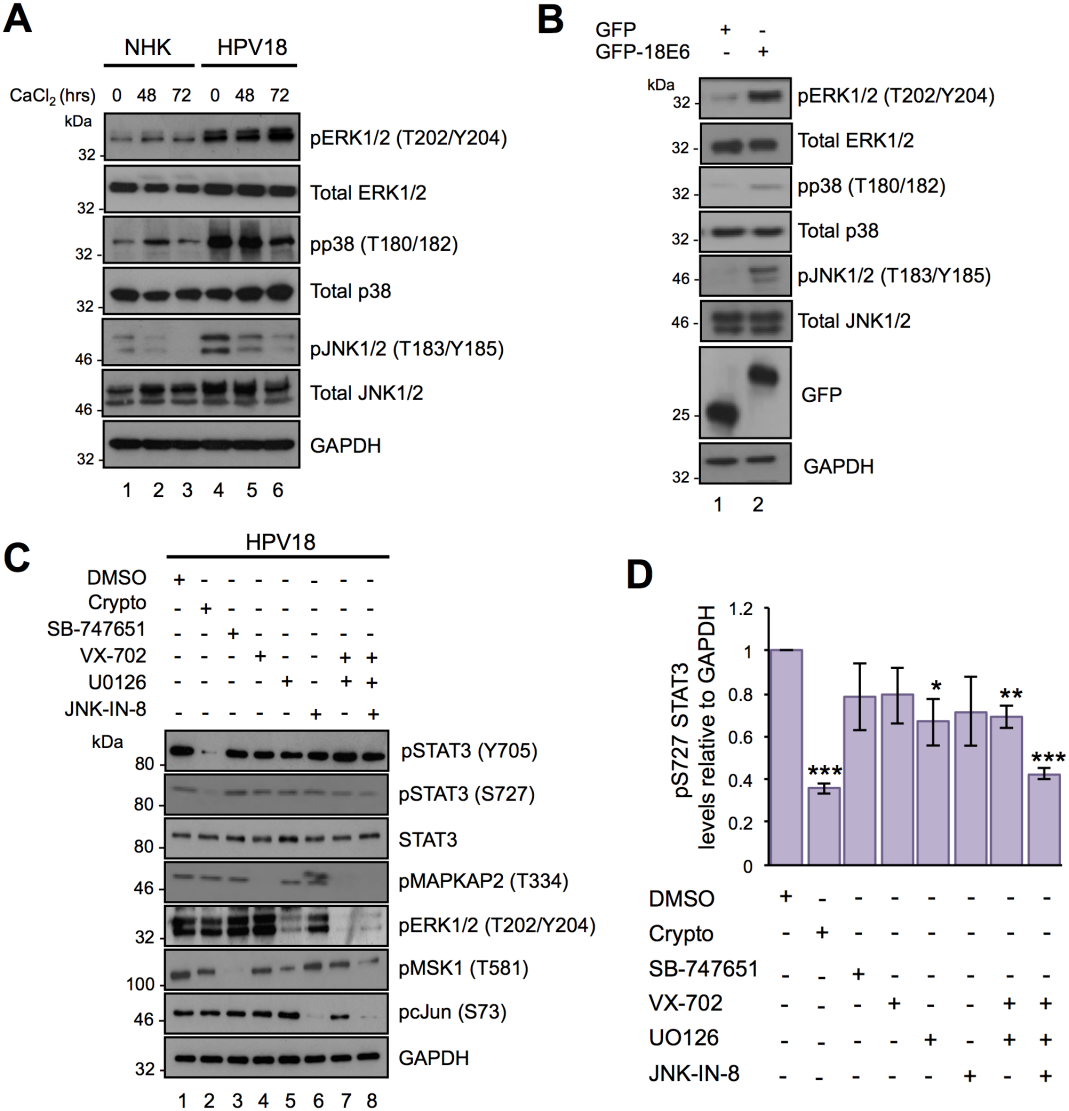
Identifying the protein kinases responsible for the S727 phosphorylation of STAT3 in HPV18 containing keratinocytes. **A)** Representative western blot of keratinocytes subjected to high calcium differentiation analysed with antibodies specific for the total and phosphorylated forms of ERK1/2 (Thr202/Tyr204), p38 (Thr180/Tyr182) and JNK (Thr183/Tyr185). GAPDH served as a loading control. Data shown are representative of at least three independent biological repeats. **B)** Representative western blot of C33a cells transfected with GFP and GFP tagged HPV18 E6 and analysed using specific antibodies detecting phosphorylated and total forms of ERK1/2 (Thr202/Tyr204), p38 (Thr180/Tyr182) and JNK (Thr183/Tyr185). GAPDH served as a loading control. **C)** Representative western blots of HPV18 containing keratinocytes incubated with specific inhibitors of STAT3 (Crypto), MSK1 (SB-747651A; SB), p38 (VX-745; VX)), JNK (JNK-IN-8), MKK1/2 (UO126) or in combination as described in the methods and materials and examined with antibodies specific for phosphorylated and total STAT3. The phosphorylation status of substrate proteins pMSK1 (T581), pcJun (S73), pMAPKAPK2 (T334), and ERK1/2 (T202/Y204) demonstrated inhibitor efficacy and specificity. **D)** Quantification of the protein band intensities of (C) standardised to GAPDH and shown relative to the DMSO control. Bars represent the means ± standard deviation from at least three independent biological repeats. *P<0.05, **P<0.01, ***P<0.001 (Student’s t-test).

A number of candidate STAT3 S727 kinases have emerged including; CDK5 [46], mTOR [47], NEMO-like kinase (NLK) [48] and several PKC isoforms [49,50]. However, the strongest evidence indicates that MAPK members mediate the serine phosphorylation of STAT3 [18]. The plethora of kinases capable of phosphorylating STAT3 implies that under physiological conditions this event might be restricted by cell type or the nature of the stimulus. We therefore set out to determine which kinases were responsible for STAT3 S727 phosphorylation in keratinocytes harbouring HPV18 genomes. We focused on the canonical MAPK members since S727 is embedded within a strong MAPK consensus sequence (^725^PMSP^728^). To determine whether MAPK members could directly phosphorylate STAT3, purified recombinant JNK1, ERK2 and p38α were subject to an *in vitro* kinase assay by using bacterially expressed and purified STAT3 as the substrate. All three kinases successfully phosphorylated STAT3 (S3 Fig). Whilst data generated from *in vitro* kinase assays demonstrates the ability of a kinase to directly phosphorylate a substrate, it is essential to confirm that the kinase in question is active in cell culture. To this end, we tested the activation status of each of the canonical MAPK members in differentiating keratinocytes. In these studies, levels of total ERK1/2 were increased in HPV18-containing keratinocytes compared to NHK controls (Fig 5A; compare lanes 1–3 and 4–6). In accordance with a key role in keratinocyte proliferation, keratinocytes displayed detectable ERK1/2 phosphorylation [51] (Fig 5A). As expected, both basal and differentiation-induced ERK1/2 phosphorylation was greater in HPV positive cells (compare lanes 1 and 4, and lanes 3 and 6). Whereas total p38 protein expression did not alter in the presence of HPV18, the levels of p38 phosphorylation were greater in both undifferentiated and differentiated HPV18-containing keratinocytes (Fig 5A; compare lanes 1 and 4, 3 and 6). In contrast with ERK1/2 and p38, less is known of JNK1/2 regulation by HPV18; however, studies demonstrate that JNK1/2 signalling can prevent keratinocyte differentiation [52]. In keeping with this role, levels of JNK1/2 phosphorylation were highest in undifferentiated NHKs, and decreased rapidly upon differentiation in high calcium media (Fig 5A; compare lanes 1 and 3). JNK1/2 phosphorylation also declined in differentiating HPV18 positive keratinocytes but overall levels were noticeably higher than in NHK (compare lanes 1 and 4, lanes 3 and 6). Together, these data demonstrate the presence of active MAPK members in HPV18 genome-containing cells. Moreover, in transient transfection experiments the phosphorylation of all three MAPK members was increased in C33a cells expressing GFP-18E6 compared to a GFP control (Fig 5B).

To test the roles of endogenous MAPK in the regulation of STAT3, we compared the levels of STAT3 S727 phosphorylation in undifferentiated HPV18-containing cells treated with the p38 inhibitor VX-702 [53], the MKK1/2 inhibitor UO126 (prevents ERK1/2 phosphorylation and activation) [54] and the JNK1/2 inhibitor JNK-IN-8 [55], alone or in combinations (Fig 5C and 5D). The STAT3 inhibitor cryptotanshinone served as a positive control in these experiments and the Mitogen and Stress activated protein Kinase (MSK) inhibitor SB-747651 acted as a negative control, given that in our tests MSK did not phosphorylate STAT3 *in vitro* (S3 Fig). As shown in Fig 5C, STAT3 S727 and Y705 phosphorylation were significantly (p<0.001) impaired by cryptotanshinone (lane 2) but unaffected by the MSK inhibitor SB-747651 (lane 3). STAT3 Y705 phosphorylation remained intact in cells treated with the MAPK inhibitors, indicating that tyrosine phosphorylation of STAT3 is not dependent on active MAPK in keratinocytes. STAT3 S727 phosphorylation was largely unaffected in cells treated with VX-702 (p38 inhibitor) and JNK-IN-8, and was only partially suppressed by the MKK1/2 inhibitor UO126 (lanes 5 (p<0.05) and 7 (p<0.01)). Importantly, the combination of UO126, VX-702 and JNK-IN-8, blocking all three MAPK members, was required to significantly (p<0.001) reduce STAT3 S727 phosphorylation (lane 8) (Fig 5D). Western blot analysis of the phosphorylated forms of the MAPK substrates MAPKAP-K2 (p38), MSK (ERK1/2) and c-Jun (JNK1/2) were used to demonstrate the efficacy of inhibitor treatment. These data indicate that several MAPK family members can phosphorylate STAT3 S727 in HPV18-positive keratinocytes.

### Active STAT3 is required for viral gene expression in undifferentiated primary keratinocytes

**Fig 6:**
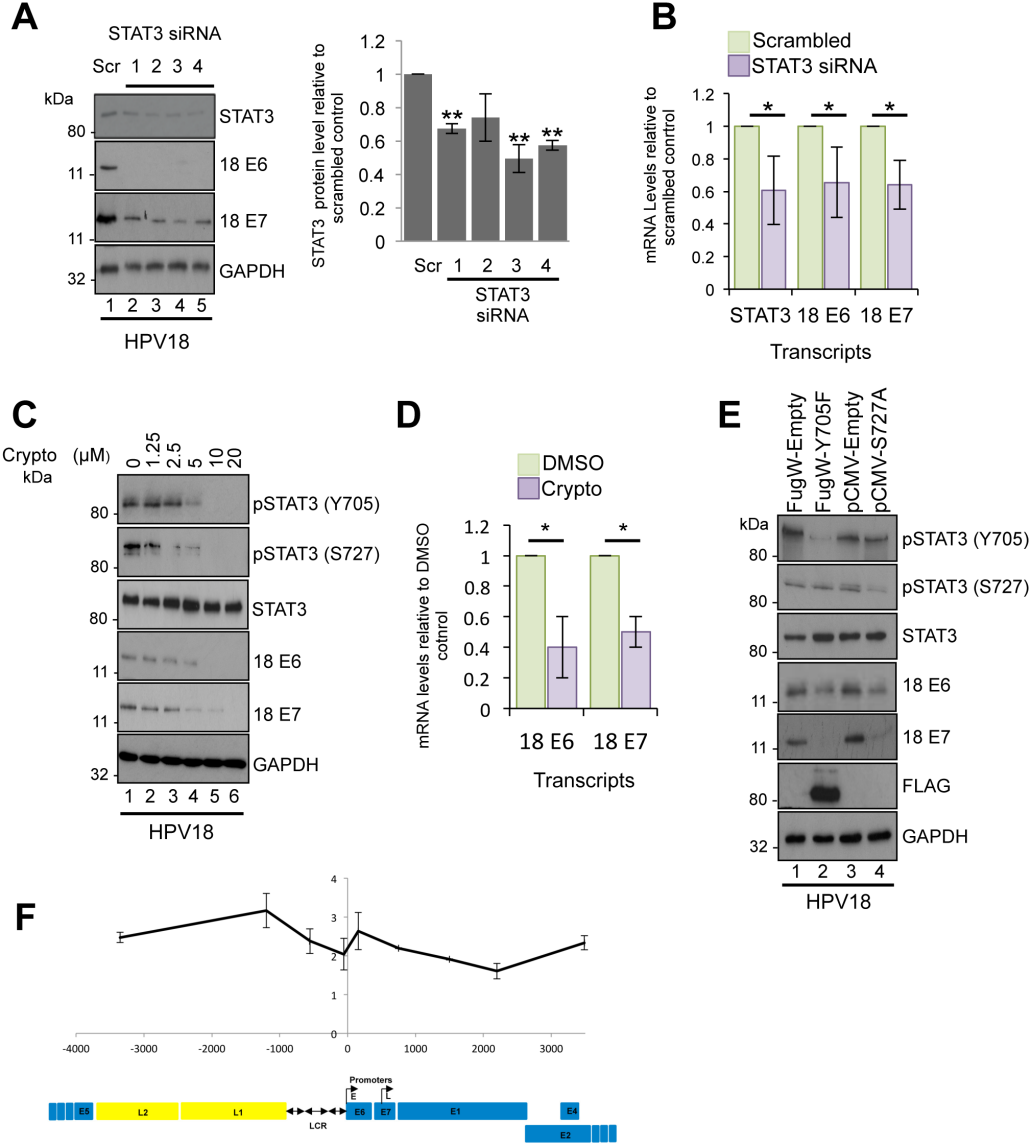
STAT3 is required for viral gene expression in undifferentiated keratinocytes. **A)** Left: Representative western blots of HPV18 containing keratinocytes transfected with a panel of 4 STAT3 specific siRNA and a scrambled control and analysed by western blotting using a total STAT3 antibody and antibodies detecting the E6 and E7 HPV18 early proteins. GAPDH served as loading control. Right: Quantification of the STAT3 protein band intensities standardised to GAPDH levels. Data are expressed as fold reduced compared to scramble siRNA treated cells. Bars represent the means ± standard deviation of at least three biological repeats. **p <0.01 (Student’s t-test). **B)** Samples treated as in (A) and RNA extracted for qRT-PCR determination of the *stat3* and HPV early gene expression levels. Samples were normalized against U6 mRNA levels and data are presented relative to the scrambled control. Bars represent means ± standard deviation of at least three biological repeats. *P<0.05 (Student’s t-test). **C)** Representative western blots from HPV18 containing keratinocytes treated with increasing doses of the STAT3 inhibitor cryptotanshinone and analysed with antibodies specific for phosphorylated and total STAT3, and E6 and E7 HPV18 early proteins. GAPDH served as a loading control. Data are representative of at least three biological repeats. **D)** RNA was extracted from HPV18 containing keratinocytes treated with 10 μM cryptotanshinone and qRT-PCR analysis performed for levels of HPV E6 and E7 mRNA. Samples were normalized against the U6 mRNA levels and expressed relative to the DMSO control. Bars represent means ± standard deviation of at least three biological repeats. *P<0.05 (Student’s t-test). **E)** Representative western blots from HPV18 containing keratinocytes transduced with a lentivirus encoding a dominant negative Y705F phospho-site mutant or transiently transfected with a dominant negative STAT3 S727A mutant and analysed by blotting with antibodies specific for phosphorylated and total STAT3 and the E6 and E7 HPV18 early proteins. An antibody detecting the FLAG epitope confirmed expression of the Y705F STAT3 mutant and GAPDH served as a loading control. Data shown are representative of at least three biological repeats. **F)** Association of STAT3 with HPV18 genomes was assessed in HPV18 containing keratinocytes by ChIP with control antibody (rabbit IgG) or STAT3-specific antibody. Co-precipitating DNA was analysed by qPCR. The x axis represents the position in the HPV genome amplified and each data point represents the central point in each amplicon. Enrichment of STAT3 binding is expressed as fold over IgG negative control calculated by the ΔΔC_T_ method. The data represent the mean and standard deviation of three independent experiments. A graphical representation of the HPV18 genome is shown below the data, linearized for ease of presentation (E; early promoter, L; late promoter, LCR; long control region).

Given that STAT3 activation and the expression of STAT3-dependent genes were increased in HPV18-positive cells, we assessed if STAT3 was necessary for the virus life cycle. For this, STAT3 was depleted from primary keratinocytes harbouring HPV18 genomes using a panel of four commercially validated siRNAs. Each siRNA produced a reproducible depletion of STAT3 by an average of 38% compared to a scrambled control, and did not reduce levels of the STAT5 protein (Fig 6A and S2B Fig). Despite the modest depletion of STAT3, expression of the HPV18 E6 and E7 proteins was greatly reduced compared to the scrambled control (Fig 6A). To assess if the inhibition of HPV18 oncoprotein expression occurred at the level of transcription, HPV18-positive keratinocytes were transfected with a pool of the STAT3 siRNA, and total RNA isolated and assayed by qRT-PCR for the levels of STAT3, E6 and E7 transcripts. STAT3 depletion (p<0.05) caused a significant decrease in early transcript expression (E6 p<0.05, E7 p<0.05), indicating that STAT3 has a role in HPV gene expression in undifferentiated cells (Fig 6B).

To rule out the possibility that siRNA studies perturbed a crucial STAT3 protein scaffolding function [48], we used small molecule inhibitors to specifically block STAT3 phosphorylation and transactivation whilst retaining levels of total STAT3 protein. For this, we monitored the level of HPV protein expression in undifferentiated HPV containing keratinocytes treated with increasing concentrations of the STAT3 inhibitor cryptotanshinone. As shown in Fig 6C, inhibitor treatment reduced STAT3 phosphorylation in a dose dependent manner without affecting total STAT3 levels. Importantly, cryptotanshinone treatment had no adverse effect upon keratinocyte viability during the term of the experiment (S4 Fig). Concentrations of cryptotanshinone that blocked STAT3 phosphorylation also decreased E6 and E7 protein (Fig 6C) and mRNA transcript levels (p<0.05) (Fig 6D).

Next, we wished to demonstrate the contribution of the individual Y705 and S727 phosphorylation events to STAT3 regulation of HPV oncogene expression. This was important given that an emphasis has been placed on Y705 phosphorylation as a critical step in STAT3 activation; however, under certain circumstances S727 phosphorylation is necessary for full STAT3 transactivation [18]. Dominant negative forms of STAT3 were used in which Y705 was replaced with a phenylalanine or S727 substituted with an alanine, generating mutants with a single unphosphorylatable site [56,57]. The STAT3 mutants were expressed in HPV18-containing keratinocytes, and over-expression of the Y705F mutant confirmed by western blot with an anti-FLAG antibody (the pFugW-Y705F plasmid encodes a FLAG-epitope tagged STAT3) (Fig 6E, lane 2). Expression of the Y705F mutant corresponded with a loss of Y705 phosphorylation but had no overall impact on S727 phosphorylation (Fig 6E, compare lanes 1 and 2). Likewise, expression of S727A ablated the observed S727 phosphorylation but did not reduce Y705 phosphorylation (Fig 6E, compare lanes 3 and 4). Over-expression of the phosphorylation site mutants had negligible impact on STAT5 phosphorylation (S2C Fig). Notably, expression of either of the phosphorylation site mutants led to a substantial reduction in HPV18 E6 and E7 protein expression (Fig 6E). Taken together, these data demonstrate that STAT3 phosphorylation at both Y705 and S727 is essential for HPV oncogene expression in undifferentiated keratinocytes.

We hypothesized that STAT3 might control HPV early gene expression by binding directly to the HPV genome. Indeed, recent chromatin immunoprecipitation sequencing (ChIP-Seq) data observed STAT3 binding to the long control region (LCR) of integrated virus genomes in the HPV18-positive HeLa cervical cancer cell line [58]. The LCR is bound by a number of host transcription factors and is essential for driving early viral transcription [59]. We used ChIP followed by qPCR to investigate whether STAT3 bound to the LCR of HPV18 episomes in primary keratinocytes [60]. In contrast to observations in the cancer cell line, we failed to detect significant STAT3 enrichment at the LCR of HPV18 in undifferentiated cells (Fig 6F). Our combined data demonstrates a requirement for STAT3 phosphorylation and activation in HPV oncogene expression; likely independent of a direct interaction between STAT3 and the viral LCR.

### Suppression of STAT3 impairs cell cycle progression and results in loss of HPV18 genome maintenance in undifferentiated keratinocytes

**Fig 7:**
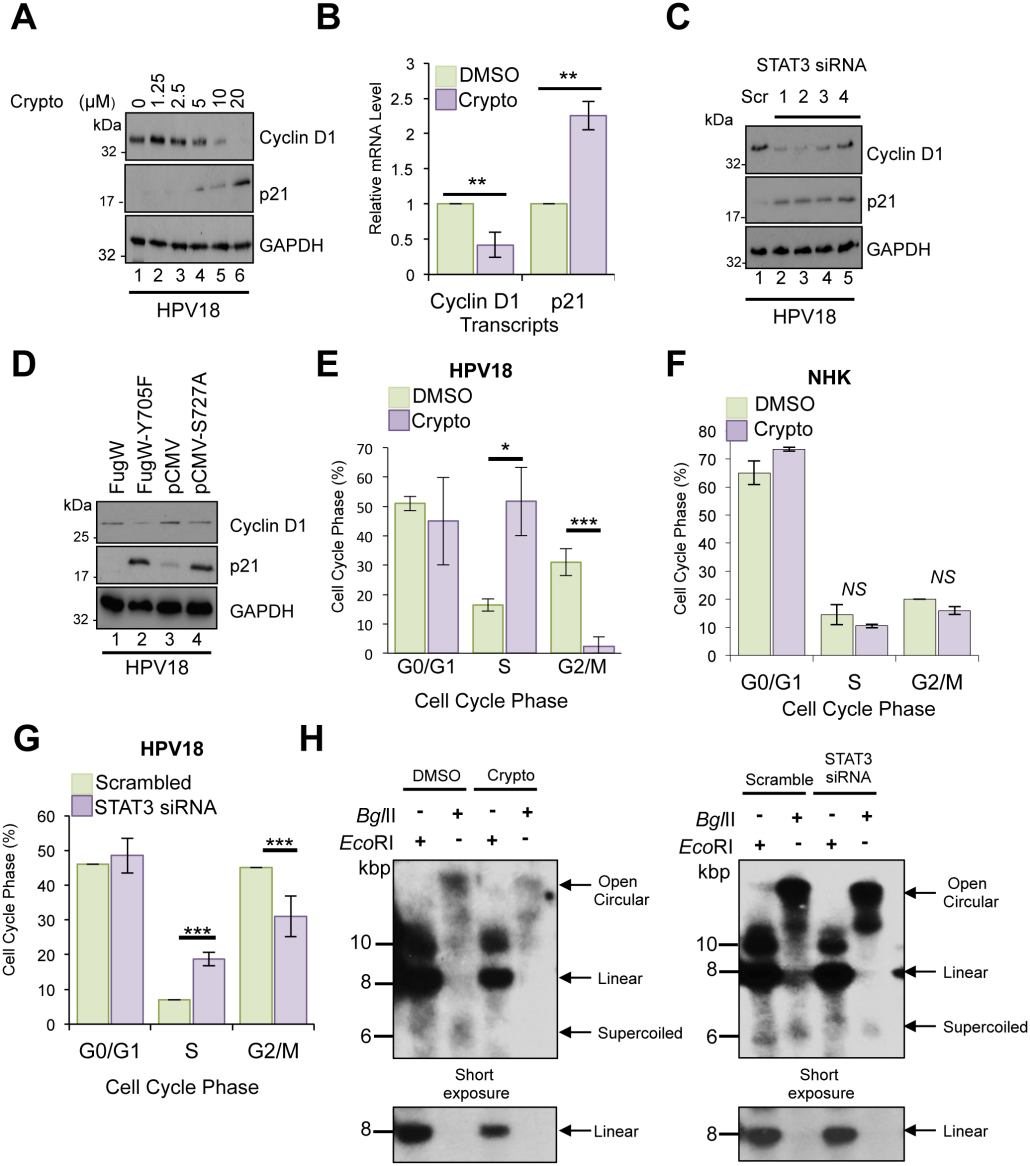
Suppression of STAT3 impairs cell cycle progression and HPV genome maintenance in undifferentiated keratinocytes. **A)** Representative western blots from HPV18 containing keratinocytes treated with increasing doses of the STAT3 inhibitor cryptotanshinone and analysed with antibodies specific for cyclin D1 and p21. GAPDH served as loading control. Data are representative of at least three biological independent repeats. **B)** RNA was extracted from HPV18 containing keratinocytes incubated with 10 μM cryptotanshinone and analysed by qRT-PCR for levels of *ccnd1* and *p21* mRNA. Samples were normalised against U6 mRNA levels and presented relative to the DMSO control. Bars represent means ± standard deviation of at least three biological repeats. **P<0.01 (Student’s t-test). **C)** Representative western blots of HPV18 containing keratinocytes transfected with a panel of 4 STAT3 specific siRNA and a scrambled control and analysed by western blotting using antibodies specific for cyclin D1 and p21. GAPDH served as loading control. Data are representative of at least three biological independent repeats. **D)** Representative western blots from HPV18 containing keratinocytes transduced with a lentivirus encoding a dominant negative Y705F phospho-site mutant or transiently transfected with a dominant negative STAT3 S727A mutant and analysed by blotting with antibodies specific for cyclin D1 and p21. GAPDH served as loading control. Data are representative of at least three biological independent repeats. Representative flow cytometric analysis of the cell cycle in **E**) HPV18 containing keratinocytes or **F)** normal human keratinocytes treated with 10 μM cryptotanshinone. **G)** As in E except cells treated with a pool of four STAT3 specific siRNAs. Data are expressed as percentage of cells at each stage of the cell cycle. Bars represent means ± standard deviation of at least three biological repeats. *P<0.05, ***P<0.001 (Student’s t-test) compared to DMSO or scrambled control cells. **H)** Representative Southern blot analysis of undifferentiated HPV18 containing keratinocytes treated with 10 μM cryptotanshinone or a pool of four STAT3 specific siRNAs.

Considering that STAT3 is well known for its proliferative effects, we asked whether STAT3 contributes towards E6 and E7 gene expression by facilitating cell proliferation. We investigated the effects of cryptotanshinone on the STAT3-dependent gene product and key cell cycle regulator, cyclin D1. Western blot analyses were performed to determine the expression of cyclin D1 in undifferentiated HPV-positive keratinocytes after 24 hours of cryptotanshinone treatment. Fig 7A showed that cryptotanshinone decreased the expression of cyclin D1 in a dose-dependent manner. Lower levels of cyclin D1 protein expression correlated with significantly (p<0.01) lower levels of cyclin D1 mRNA in these cells as confirmed by qRT-PCR (Fig 7B). In keeping with inhibitor studies, depletion of total STAT3 protein by siRNA (Fig 7C) or expression of phosphorylation site dominant negative forms of STAT3 (Fig 7D) also resulted in reduced cyclin D1 expression in HPV positive keratinocytes. STAT3 also inhibits cell cycle arrest and senescence through inhibition of the cyclin dependent kinase (CDK) inhibitor p21^WAF1/CIP1^. Suppression of p21^WAF1/CIP1^ expression is facilitated indirectly by suppression of the p53 tumour suppressor protein [61], or thought to occur directly by formation of a STAT3-cyclin D1 transcriptional repressor complex bound to the p21^WAF1/CIP1^ promoter [62]. Loss of STAT3 expression and activity led to an up-regulation of p21^WAF1/CIP1^ expression at both the protein and transcript level (Fig 7A–7D). Given that cell cycle progression depends on the sequential expression of stage-specific cyclins and the activity of their corresponding CDKs we examined the cell cycle profiles of HPV positive keratinocytes in which STAT3 activity was impaired. In the presence of cryptotanshinone, more keratinocytes were observed in S phase (p<0.05) with a concomitant decrease (p<0.001) in the fraction of cells in G2/M (Fig 7E). Of note, cryptotanshinone treatment had no impact on the cell cycle profile of normal human keratinocytes (Fig 7F). To rule out any non-specific effects of inhibitor treatment we confirmed a similar cell cycle block in HPV18-containing keratinocytes when STAT3 was depleted by siRNA (p<0.001) (Fig 7G). These observations are consistent with a delay in transit of cells from S to G2 phase upon inhibition of STAT3 in HPV18-containing cells, but not in normal human keratinocytes.

The importance of STAT3 for maintaining efficient cell cycle progression prompted us to assess whether STAT3 was necessary for stable HPV genome maintenance in undifferentiated cells. HPV containing keratinocytes were grown in undifferentiated monolayer culture and incubated with cryptotanshinone for 48 hours to impair STAT3 activity or transfected with a pool of STAT3-specific siRNA to deplete total protein. Total DNA was isolated from these cells and analysed by Southern blotting for levels of virus genomic DNA. There was a reduction in HPV episome levels when STAT3 activity was ablated by small molecule inhibitor (62%) or total STAT3 protein removed by siRNA (30%) (Fig 7H). Together, these data demonstrate a role for STAT3 in maintaining cell cycle progression and stable genome maintenance in undifferentiated keratinocytes.

### STAT3 is important for cell cycle progression and HPV18 genome amplification in differentiated keratinocytes

**Fig 8:**
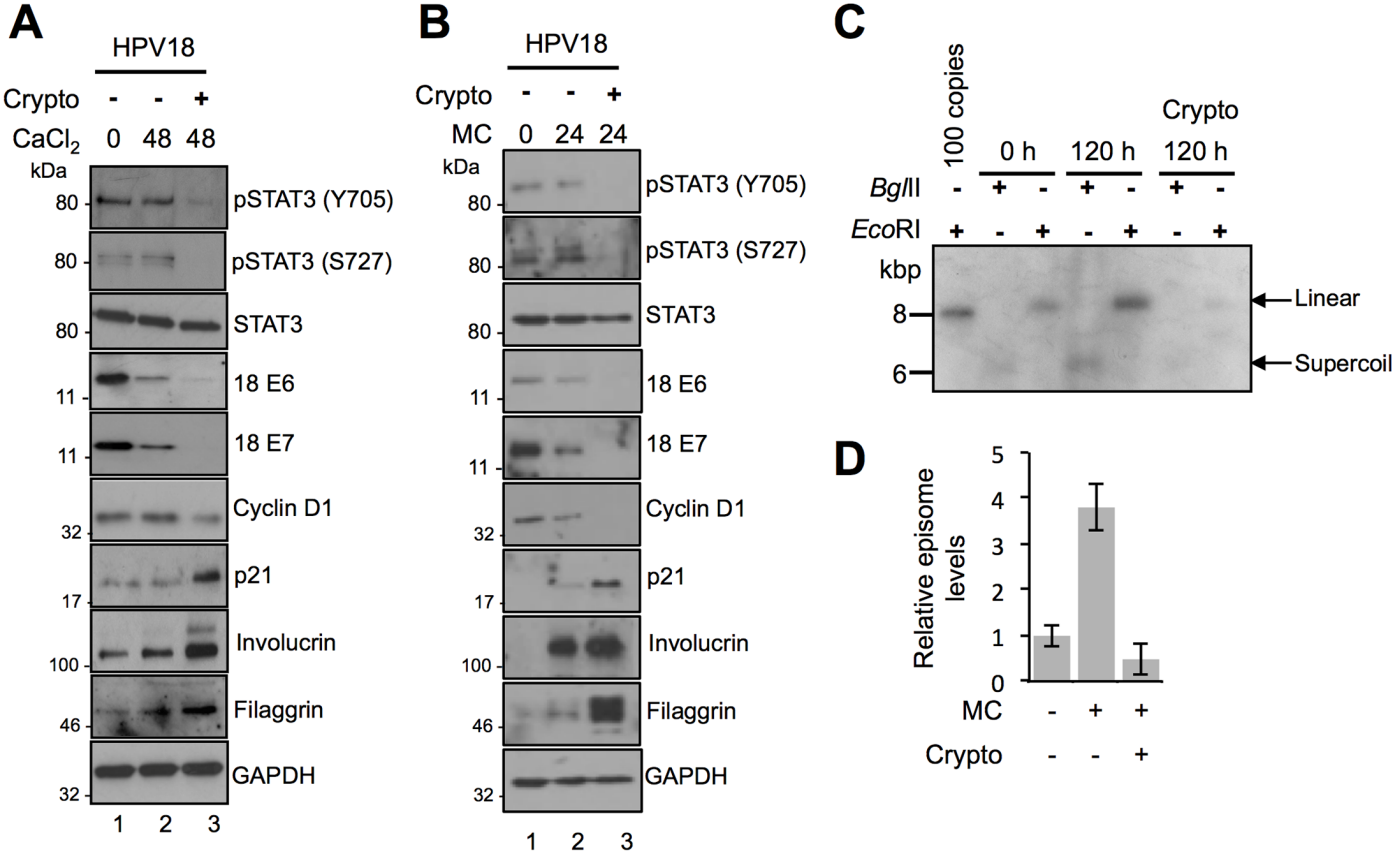
STAT3 is important for cell cycle progression and HPV18 genome amplification in differentiated keratinocytes. Representative western blots of phosphorylated and total STAT3, HPV18 E6 and E7, cyclin D1, p21, involucrin and filaggrin in **A)** calcium or **B)** methylcellulose-differentiated HPV18 containing keratinocytes in the presence or absence of 10 μM cryptotanshione. Data shown represent at least three biological repeats. **C)** Southern blot analysis of HPV18 episomes in keratinocytes treated with 10 μM cryptotanshinone and differentiated in methylcellulose for 120 hours. DNA was linearized with *Eco*RI, producing a single band running at approximately 8 kbp, demonstrating a differentiation-dependent increase in viral episome copy number in untreated control cells and a reduction in episome copy number in cells treated with cryptotanshinone. Digestion with *Bgl*II shows a lack of detectable multimeric/integrated HPV genomes in all experimental conditions. Data shown are representative of two donor cell lines. **D)** Signal intensity was quantified using ImageJ software. Bars represent means ± standard deviation of two biological repeats from two donor cell lines.

Given that levels of STAT3 phosphorylation were maintained upon keratinocyte differentiation, we wished to assess if STAT3 was also needed for the differentiation-dependent stages of the HPV18 life cycle. HPV18-containing keratinocytes were co-cultured for 48 hours in media containing high calcium to induce differentiation and cryptotanshinone to block STAT3 activation. Whilst levels of phosphorylated STAT3 and total E6 and E7 early proteins were maintained upon differentiation in control cells, they were suppressed by cryptotanshinone (Fig 8A; compare lanes 2 and 3). By maintaining high levels of cyclins, HPV-containing cells remain active in the cell cycle upon differentiation to allow for virus genome amplification. In accordance with this, upon differentiation high levels of cyclin D1 were maintained, and expression of the CDK inhibitor p21^WAF1/CIP1^ was not elevated (Fig 8A; compare lanes 1 and 2). Blockade of STAT3 activation reduced cyclin D1 levels in differentiated cells (Fig 8A; compare lanes 2 and 3), and consistent with the observed down-regulation of cell cycle pathways, cryptotanshinone increased the expression of p21^WAF1/CIP1^ (Fig 8A; compare lanes 2 and 3). The switch between keratinocyte proliferation and differentiation is tightly controlled and STAT3 has been identified as a regulator of this process [25]. Therefore, we asked whether STAT3 contributed to the suppression of keratinocyte differentiation observed during the HPV life cycle. In untreated keratinocytes harbouring HPV18 genomes, levels of the differentiation-specific proteins involucrin and filaggrin did not increase significantly upon incubation in high calcium media (Fig 8A; compare lanes 1 and 2). In contrast, cells treated with cryptotanshinone displayed enhanced expression of these differentiation-dependent proteins (Fig 8A; compare lanes 2 and 3). These experiments indicate that STAT3 regulates the cell cycle and differentiation marker expression in differentiating HPV-positive keratinocytes. To investigate if STAT3 was also important for differentiation-dependent HPV18 genome amplification, HPV18 containing cells were treated with STAT3 inhibitors and differentiated by suspension in methylcellulose prior to harvesting the DNA for Southern blotting. Western blot analysis confirmed similar effects of STAT3 inhibition on HPV18 gene expression and keratinocyte proliferation and differentiation marker expression in the methylcellulose differentiated keratinocytes (Fig 8B). Moreover, treatment with cryptotanshinone significantly reduced amplification of HPV18 genomes upon keratinocyte differentiation (Fig 8C and 8D). These data indicate that STAT3 is required for differentiation-dependent stages of the HPV life cycle.

### STAT3 is necessary for delayed differentiation and increased keratinocyte proliferation in stratified epithelia

**Fig 9:**
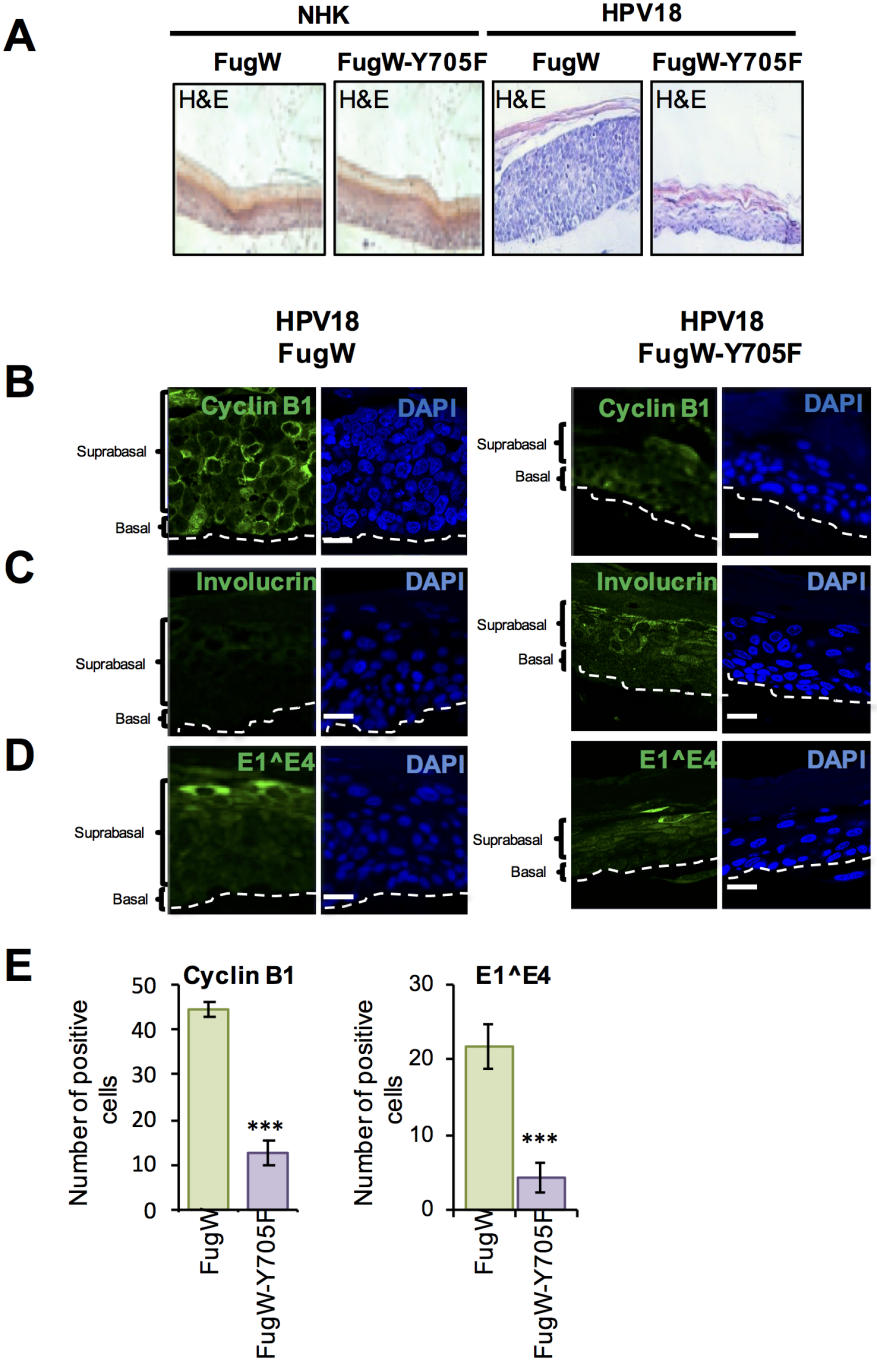
STAT3 is necessary for delayed differentiation and increased keratinocyte proliferation in a stratified epithelium. **A)** Representative organotypic raft cultures of NHK and HPV18-containing keratinocytes transduced with empty lentivirus and lentivirus expressing a dominant negative phosphorylation null Y705F mutant STAT3 fixed at day 14 and stained with haematoxylin and eosin (H&E) to assess morphology. Sections were also stained with antibodies specific for **B)** cyclin B1 **C)** involucrin and **D)** E1^E4. Nuclei are visualised with DAPI (blue) and white dotted lines indicate the basal cell layer. **E)** The number of cells positive for cyclin B1 and E1^E4 in the empty vector and Y705F STAT3 transduced HPV18-containing keratinocyte sections was counted in 5 fields of vision from sections of three independent raft cultures from two donor lines. Bars represent means ± standard deviation. ***P<0.001 (Student’s t-test) compared to empty vector control.

Our monolayer differentiation assays suggested that the ability of HPV to promote cell cycle re-entry and delay differentiation was dependent on active STAT3. To address this in the context of a fully stratified epithelium, NHK and HPV18-containing keratinocytes were transduced with a lentivirus expressing the dominant negative phosphorylation site Y705F STAT3 mutant or an empty vector control (pFugW). The Y705F mutant was utilized as it appeared marginally more effective at impairing HPV gene expression compared to the S727A mutant (Fig 6D and 6E). Forty-eight hours after transduction cells were seeded onto collagen plugs and grown as organotypic raft cultures, and sections stained with haematoxylin and eosin (Fig 9A). In comparison to NHK epithelium, the presence of HPV18 genomes was associated with a thickening of the parabasal and spinous cell layers as previously shown [13]. Whilst the over-expression of a dominant negative STAT3 protein had no impact on the morphology of NHK rafts, the overall thickness of rafts produced from HPV18-containing cells expressing STAT3-Y705F was consistently reduced, and their appearance more closely resembled the morphology of the structures produced from the normal donor cells (higher magnification image in S5A Fig).

Amplification of the viral genome occurs in suprabasal cells that have entered a protracted G2 phase of the cell cycle, and these cells can be identified by accumulation of cytoplasmic cyclin B1 [63]. Thus, to assess cell cycle status of suprabasal cells, rafts were stained for cyclin B1. In NHK, cyclin B1 was only observed in the basal layers of the raft and this was unaffected by transduction with the Y705F STAT3 mutant (S5B Fig). In contrast, we observed a significant (p<0.001) decrease in the population of cyclin B1 positive suprabasal cells following staining of the Y705F transduced HPV18 rafts, relative to empty vector transduced HPV18 rafts (Fig 9B and 9E). These data illustrate the failure of cells lacking active STAT3 to maintain cell cycle activity in the upper stratified layers.

Immunofluorescent staining of the differentiation marker involucrin was used to assess the differentiation state of the rafts. As an intermediate stage differentiation marker, involucrin is found in the suprabasal compartment of a normal epithelium (S5C Fig). Empty vector transduced HPV18 rafts showed a delayed staining pattern, with expression restricted to the upper suprabasal layers of the epithelium (Fig 9C). In contrast, abundant involucrin staining was noted throughout the spinuous layers of the epithelium in rafts transduced with the dominant negative Y705F phosphorylation mutant. Sections were also stained for the viral protein E1^E4, a marker of productive infection [32,64]. E1^E4 staining was observed in the mid and upper suprabasal layers of HPV18 control rafts, but was significantly (p<0.001) reduced in rafts expressing STAT3 Y705F (Fig 9D and 9E; a wide field image available in S5D Fig). Taken together, these data indicate that active STAT3 is dispensable for the formation of a stratified epithelium in NHK, but is necessary for increased suprabasal cellular proliferation and cell cycle progression in cells harbouring HPV18 genomes. STAT3 function also contributes to the delay in differentiation and expression virus proteins observed in HPV-positive cells.

### STAT3 expression and phosphorylation positively correlates with disease state in cervical cancer samples

**Fig 10:**
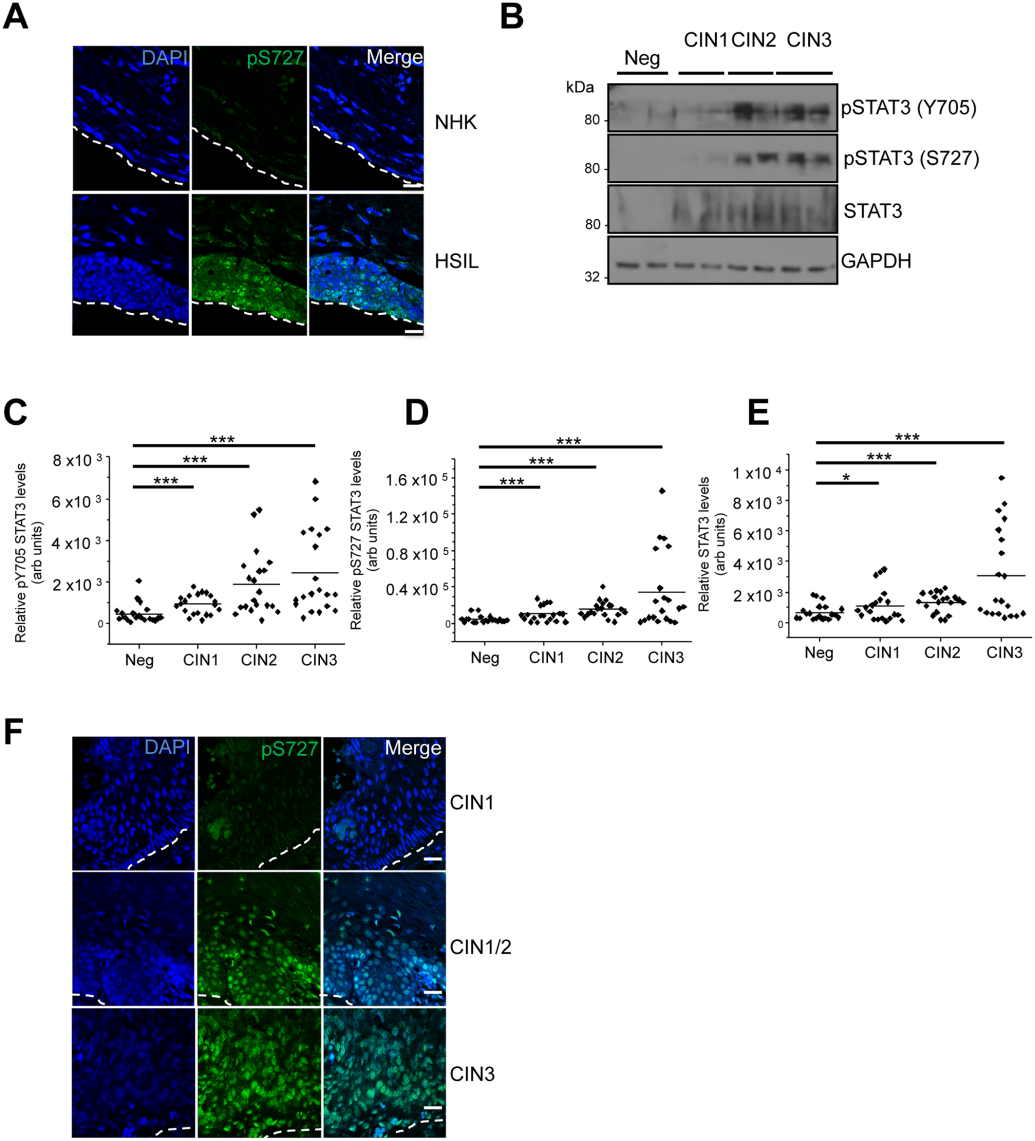
STAT3 expression and phosphorylation is increased in HPV-associated cervical disease. **A)** Representative immunofluorescence analysis of sections from organotypic raft cultures of NHK and a W12 cell line presenting with HSIL morphology detecting pS727 STAT3 levels (green). Nuclei were visualized with DAPI (blue) and the white dotted line indicates the basal layer. Images were acquired with identical exposure times. **B)** Representative western blots from cytology samples of CIN lesions of increasing grade analysed with antibodies specific for phosphorylated (Y705 and S727) and total STAT3 levels. GAPDH served as a loading control. **C-E)** Scatter dot plot of densitometry analysis of a panel of cytology samples. 20 samples from each clinical grade (neg, CIN I-III) were analysed by western blot and densitometry analysis was performed using ImageJ. Phosphorylated STAT3 levels were first normalised against total STAT3 levels before normalising against protein levels using GAPDH as a loading control. **F)** Representative immunofluorescence analysis of tissue sections from cervical lesions of increasing CIN grade. Sections were stained for pS727 STAT3 levels (green) and nuclei were visualized with DAPI (blue). Images were acquired with identical exposure times. Scale bar, 20 μm.

Although STAT3 phosphorylation was increased in primary keratinocytes containing HPV18, we queried whether aberrant STAT3 phosphorylation correlated with cervical cancer initiation and progression. Initially, we utilized the W12 *in vitro* model system [65]. The W12 system was developed from a polyclonal culture of cervical squamous cells naturally infected with HPV16, derived by explant from a low-grade squamous intraepithelial lesion (LSIL). At early passages these cells recapitulate an LSIL in organotypic raft cultures. However, following long-term culture these cells mirror the events associated with cervical cancer progression, with phenotypic progression to high-grade intraepithelial lesions (HSIL) and squamous cell carcinoma. Organotypic raft cultures were generated from NHK cells and a W12 clone representing a HSIL phenotype and stained for STAT3 S727 phosphorylation. In contrast to the NHK control, high levels of S727 phosphorylation was observed in the nuclei of cells throughout the basal and suprabasal layers of the HSIL raft (Fig 10A). These data confirm that STAT3 S727 phosphorylation is greater in a high-grade lesion compared to NHK control cells. They also confirm that increased STAT3 S727 phosphorylation is observed in the context of a high-risk HPV16 infection.

Next, cervical liquid based cytology samples from a cohort of HPV16 positive patients representing the progression of disease development (CIN1-CIN3) and HPV negative normal cervical tissue controls were collected from the Scottish HPV archive and examined for the levels of STAT3 protein and phosphorylated STAT3. Western blot analysis of the samples showed that in normal cervical tissue STAT3 expression and phosphorylation was low (Fig 10B). However, STAT3 expression and its phosphorylation increased in the disease samples and this significantly correlated with the degree of disease severity (Fig 10B–10E). Finally, we used immunofluorescence to examine phosphorylated STAT3 S727 levels in a series of human cervical sections classified as LSIL (CIN1), LSIL with foci of HSIL (CIN1/CIN2) and CIN3. An analysis of the staining pattern showed a clear difference between the three groups (Fig 10F), with greater STAT3 S727 phosphorylation in the high-grade neoplasia compared with lower grade lesions. Together, these findings demonstrate that STAT3 phosphorylation is increased in HPV positive keratinocytes including those of a natural HPV infection and correlate with cervical disease progression.

## Discussion

This study identifies the STAT3 transcription factor as a critical regulator of the HPV life cycle. Using calcium and methylcellulose-mediated differentiation in monolayer, and organotypic raft culture models of primary human keratinocytes harbouring HPV18 episomes, we discovered that STAT3 was necessary for HPV18 gene expression and viral DNA replication in undifferentiated cells and in stratified epithelium. In undifferentiated cells, a loss of STAT3 transcription factor activity—brought about through the use of small molecule inhibitors or expression of dominant negative STAT3 phosphorylation mutant proteins—led to a reduction in HPV oncogene expression. This required the transcription factor function of STAT3, but it did not entail direct binding of STAT3 to the viral LCR. Our findings are in contrast to ChIP-Seq studies gathered from the HPV18-positive HeLa cervical cancer cells. This may in part be due to differences in STAT3 expression levels between primary keratinocytes and cancer cell lines. It is also possible that additional changes to the host cell signalling machinery or the integration of the viral genome, allow for STAT3 binding. The mechanism by which STAT3 activates HPV gene expression is therefore likely to be indirect. One possible mechanism would be to generate a cellular milieu favourable for virus transcription. In this regard, STAT3 is a driver of cell cycle progression and keratinocyte proliferation. Our results show that undifferentiated HPV-containing keratinocytes express cyclin D1 and this is largely STAT3-dependent as STAT3 inhibitor treatment, or depletion by siRNA resulted in a loss of proliferative marker expression and an increase in expression of the cell cycle arrest protein p21^WAF1/CIP1^. Furthermore, these changes correlated with induction of S phase arrest. This likely results from the reduced expression of cyclin D1, which in actively cycling cells must be increased in order for cells to transition into G2. A similar S-phase arrest is observed in Epstein Barr virus infected lymphoblastoid cell lines (LCLs), where STAT3 is required to relax the intra-S phase checkpoint [66]. Intriguingly, STAT3 inhibition had no discernable impact on the cell cycle profile of normal human keratinocytes, indicating that STAT3 is non-essential in HPV negative cells.

STAT3 regulates the stable maintenance of HPV episomes in undifferentiated cells and inhibition of STAT3 transcription factor activity reduces stable episome copy numbers. This can also be explained by the change in cellular proteins brought about by blockade of STAT3 transcriptional activity, which might deprive the virus of host factors essential for genome maintenance. The loss of HPV early protein expression associated with inhibition of STAT3 activity is also likely to contribute to the deficit in virus transcription and genome maintenance. Both E6 and E7 have been shown to be critical for cell cycle progression in keratinocytes and loss of either protein impairs genome maintenance [15]. In particular, the ability of E6 to stimulate the degradation of the p53 tumour suppressor protein is important for episomal maintenance in undifferentiated keratinocytes [13,67]. We observed an increase in p53 expression in cells lacking E6 expression and also noted a significant up-regulation of the p53 target gene product p21^WAF1/CIP1^. Whilst p21^WAF1/CIP1^ expression is negatively regulated by STAT3, it is plausible that p53 contributes to the increases observed in cells lacking active STAT3. Finally, there may be additional yet to be defined, viral or cellular proteins that are regulated by STAT3, which are important for episomal maintenance.

Since STAT3 is important for regulating stem cell like proliferative abilities along with controlling differentiation [24,68], we investigated whether STAT3 played a role in these properties during the HPV life cycle. As expected, our results show that HPV18-containing keratinocytes are differentiation resistant and retain their proliferative potential. This enhanced capacity is largely dependent on STAT3 as inhibitors of transcription factor activity or dominant negative STAT3 phosphorylation mutants reduced cyclin D1 expression in monolayer culture. Consistent with this notion, loss of STAT3 activity imparted a reduction in the proliferative capacity of a HPV-containing stratified epithelium resulting in increased expression of keratinocyte differentiation markers. Rafts expressing the dominant negative STAT3 protein retained the ability to stratify but were significantly altered in the expression of cell cycle regulators and differentiation markers. In particular, suprabasal cells were no longer active in the cell cycle as judged by the reduction in cyclin B1 staining. As HPV genome amplification is reliant on a prolonged G2 phase of the cell cycle, the loss of suprabasal cyclin B1 protein expression might account for the reduction in HPV genome amplification upon differentiation in cells lacking active STAT3 [63,69]. Alternatively, whilst poorly characterised, both E6 and STAT3 expression have been linked to the activation of the ATM-dependent DNA damage response, a key player in HPV DNA replication [70,71]. It would be interesting to determine whether E6-mediated activation of STAT3 might contribute to this key process.

Despite the loss of hyperplasia in the HPV18-containing rafts, the overall morphogenesis of the epithelium appeared intact and reminiscent of a normal epithelium. This suggests that whilst STAT3 phosphorylation was essential for increased proliferation, it was dispensable for normal skin development. The minimal impact on raft morphology observed in normal keratinocytes expressing the dominant negative STAT3 protein further illustrates that STAT3 appears not to be required for normal epithelial stratification. These results are in accordance with studies using gene-targeted mice containing a keratinocyte specific STAT3 knockout. These mice display no overt gross morphological differences in epithelial development but are profoundly impaired in their capacity to undergo effective wound healing in response to injury [27]. In contrast, mice engineered to express a constitutively active STAT3 in the proliferating epithelial compartment exhibit hyperplasia and a perturbed differentiation programme [28]. They are also predisposed to develop skin malignancies. Our findings clearly suggest that active STAT3 is necessary for suprabasal proliferation.

Our studies show that HPV infection leads to enhanced STAT3 Y705 and S727 phosphorylation that are maintained at high levels during keratinocyte differentiation without an alteration to the total levels of STAT3. Interestingly, we demonstrated that expression of all three HPV oncoproteins could induce STAT3 phosphorylation to a certain degree, but only E6 was sufficient to induce the dual phosphorylation and activation of STAT3. This is consistent with previous observations showing increased STAT3 Y705 phosphorylation in HPV-positive cancer cell lines, and leading to increased levels of STAT3-dependent gene products [43,72]. A number of cellular pathways converge to phosphorylate and activate STAT3 [18,20]. Conventional dogma for STAT3 activation implicates Y705 phosphorylation as necessary and sufficient to activate STAT3 in response to a number of stimuli [18]. In addition, phosphorylation of S727 within the transactivation domain of STAT3 has also been documented to activate STAT3 signalling under some conditions. Using phosphorylation null dominant negative STAT3 proteins our results clearly demonstrate that mono-phosphorylation of either site is insufficient to activate the STAT3 dependent genes analysed in this study within keratinocytes harbouring HPV and that dual phosphorylation is essential for the virus life cycle. Moreover, our work does not support previous findings that S727 phosphorylation is detrimental to Y705 phosphorylation [73]. Rather, we suggest a model of interdependent phosphorylation between Y705 and S727 sites, in which mutation of one site does not impede the phosphorylation of the other. Our work also sheds light on the identity of the host kinases that phosphorylate these sites in HPV containing primary keratinocytes. Using validated pharmacological inhibitors we demonstrate that Y705 phosphorylation is critically dependent on JAK2 activity. Whilst JAK kinases are the best characterised mediators of Y705 phosphorylation in other cellular systems, to the best of our knowledge no study has shown that E6 up-regulates their activity. Since they function downstream of growth factor and gp130 cytokine receptor pathways, it is possible that E6 up-regulates an upstream component which subsequently activates JAK2 to phosphorylate STAT3 Y705. In this regard, E6 activates the EGF receptor and increases IL-6 and oncostatin-M expression, which mediate their effects via gp130 receptors [74–76]. On-going experiments are testing whether these pathways contribute to STAT3 phosphorylation in primary keratinocytes. The mechanisms that mediate S727 phosphorylation are less clear due to a wide field of potential candidate kinases. As a first step we focused on MAPK proteins, given the presence of a strong consensus motif adjacent to S727. We found that similar to what has been reported for p38 MAPK, the phosphorylation of all three canonical MAPK proteins (ERK1/2, p38 and JNK1/2) is dysregulated in HPV containing cells [77]. Our studies suggest that inhibition of all three MAPK members is required to fully impair STAT3 S727 phosphorylation. Given that E6 can activate all three kinases, our results suggest that functional redundancy exists between the different MAPK members, providing an opportunity for S727 phosphorylation throughout differentiation. Whilst E6AP-mediated p53 degradation and PDZ domain binding are well-characterised attributes of high-risk E6 proteins, neither of these functions was required for the increase in STAT3 phosphorylation. Studies are now uncovering a wealth of additional E6 binding partners and the functional consequences of these interactions [78,79]. Use of further E6 mutants defective for binding to additional cellular targets will allow further investigation of the molecular basis for activation of these pathways.

Although the crucial role of STAT3 in both tumour cells and the tumour microenvironment is evident, gaps remain in our understanding of the regulation of STAT3 signalling in cancer. In particular, how S727 contributes to cancer development has not been fully elucidated. Given the crucial role of phosphorylation of this site for HPV containing keratinocyte proliferation, we further investigated STAT3 protein expression and phosphorylation levels in HPV associated cancers. A clear trend was evident showing an abundance of STAT3 protein and increased levels of STAT3 phosphorylation in high-grade HPV lesions compared to low-grade lesions, or control cervical samples. Moreover, staining showed obvious STAT3 S727 nuclear localization in high-grade lesions, suggesting active STAT3. Whilst STAT3 inhibitors are not yet clinically available, it might be possible to treat HPV-associated malignancies either by targeting STAT3 directly or an upstream target such as JAK2. Overall, these studies demonstrate that in addition to being activated in HPV-associated cancers, STAT3 is critically important for the productive HPV life cycle and a possible target for therapeutic intervention.

## Methods and materials

### Ethics statement

Neonate foreskin tissues were obtained from local General Practice surgeries and foreskin keratinocytes isolated in S. Roberts’ laboratory under ethical approval no. 06/Q1702/45.

### Inhibitors

The STAT3 inhibitor S3I-201 was purchased from AdooQ BioSciences and used at a final concentration of 10 μM. This cell permeable compound binds to the STAT3 SH2 domain to prevent phosphorylation and activation. Cryptotanshinone was purchased from LKT Laboratories and used at a final concentration of 10 μM to inhibit STAT3 dimerisation and activation. The STAT5 inhibitor Pimozide was purchased from Calbiochem and used at a final concentration of 10 μM, as previously described [37]. UO126 is a selective MKK1/2 inhibitor, and is used to inhibit activation of ERK1/2. It was added to cells at a final concentration of 20 μM and purchased from Calbiochem [80]. VX-702 was purchased from Tocris and used to inhibit p38 kinase activity. It is highly specific and used at a final concentration in cells of 10 μM [53]. The JNK1/2 inhibitor JNK-IN-8 was purchased from Cambridge BioSciences and used at a final concentration of 3 μM in cells [55]. SB-747651 was used to inhibit MSK1/2 and used at a final concentration of 5 μM in cells [81]. The JAK1/2 inhibitor Ruxolitinib, and JAK2 inhibitor Fedratinib were kindly provided by Dr Edwin Chen, University of Leeds and used at a final concentration of 10 μM [44]. All compounds were used at concentrations required to minimise potential off-target activity.

### Plasmids and siRNAs

Plasmids expressing HPV16 oncoproteins fused to an amino-terminal GFP protein were previously described [82,83]. GFP18 E5 was previously described [83]. E6 and E7 sequences were amplified from the HPV18 genome and cloned into peGFP-C1 with *Sal*I and *Xma*I and *Sal*I and *Bam*HI restriction enzymes. Mutations were engineered into HPV18 E6 to disable interactions with E6AP, p53 and PDZ domain containing proteins. A single point mutation was introduced to create HPV18 E6 F4V and a C-terminal truncation lacking the final four amino acids created HPV18 E6 ΔPDZ. These mutants were generated using Q5 DNA polymerase and cloned into peGFP using *Sal*I and *Xma*I. The L52A mutagenesis was performed by Genewiz (New Jersey, USA) and cloned into peGFP using *EcoRI* and *BamHI* restriction sites. The plasmids driving Firefly luciferase from the β-casein promoter and a constitutive Renilla luciferase reporter (pRLTK) were previously described [34]. pRc/CMV-STAT3 S727A (8708) [56], pcFugW (14883), pcFugW-EF.STAT3DN.Ubc.GFP (24984) [57] and pGL3-Pom^C^ (17553) [35] were purchased from Addgene (Cambridge, MA, USA) and we thank the principle investigators Jim Darnell, David Baltimore, Linzhao Cheng and Domencino Accili for depositing them.

### Cell transfection

The transfection of primary human foreskin keratinocytes (NHK) isolated from neonate foreskin tissues (ethical approval no. 06/Q1702/45) was performed in S. Roberts’ laboratory as described previously [13]. Briefly, plasmids containing the HPV18 genome were digested with *Eco*RI to release the genome, which was then re-circularised with T4 DNA ligase. The genomes were co-transfected with a plasmid encoding resistance to neomycin into low passage NHK in serum free medium. One day later, the cells were harvested and seeded onto a layer of γ-irradiated J2-3T3 fibroblasts and selected with G418 in complete E media containing foetal calf serum (FCS, Lonza) and epidermal growth factor (EGF, BD BioSciences) for 8 days. Cell colonies were pooled and expanded on γ-irradiated J2-3T3 fibroblasts. To account for donor-specific effects, NHK from two donors were used.

### Organotypic raft cultures

Keratinocytes containing wild type HPV18 genomes or the mutant E6ΔPDZ genome were grown in organotypic raft cultures by seeding the keratinocytes onto collagen beds containing J2-3T3 fibroblasts [13]. Once confluent the collagen beds were transferred onto metal grids and fed from below with FCS-containing E media without EGF. The cells were allowed to stratify for 14 days before fixing with 4% formaldehyde. The rafts were paraffin-embedded and 4 μm tissue sections prepared (Propath UK, Ltd., Hereford, UK).

For analysis of Phospho-STAT3 (S727) (ab32143, abcam), Total STAT3 (C-20: sc-482, Santa Cruz Biotechnology and 9132, CST), involucrin (SY5, Santa Cruz Biotechnology) and HPV18 E1^E4 (mouse monoclonal antibody 1D11 [84]) expression, the formaldehyde-fixed raft sections were treated with the sodium citrate method of antigen retrieval. Briefly, sections were boiled in 10 mM sodium citrate with 0.05% Tween-20 for 10 minutes. Sections were incubated with appropriate antibodies and immune complexes visualized by using Alexa 488 and 594 secondary antibodies (Invitrogen). The nuclei were counterstained with the DNA stain 4’,6-diamidino-2-phenylindole (DAPI) and mounted in Prolong Gold (Invitrogen).

### Southern blot analysis

Total genomic DNA was extracted from cell culture by phenol chloroform extraction and analysed on a 0.8% agarose gel and DNA transferred to GeneScreen nylon membrane. Complete HPV18 genome was released from the pGEMII backbone vector by *Eco*RI digestion, purified and labelled with [α-^32^P]-CTP. The membrane was incubated with this radiolabelled linear probe at 42°C overnight. Following washing the membrane was exposed to auto-radiograph film [13].

### Monolayer differentiation assays

Untransfected NHK and HPV18 containing keratinocytes were grown in complete E media until 90% confluent. Media were changed to serum free keratinocyte media without supplements (SFM medium, Invitrogen) containing 1.8 mM calcium chloride. Cells were maintained in this media for between 48–72 hours before lysis and analysis. Alternatively, keratinocytes were resuspended in E-Media containing 1.5% methylcellulose and cultured for 120 hrs prior to harvesting.

### Transfections and mammalian cell lysis

Transient transfections were performed with a DNA to Lipofectamine 2000 (ThermoFischer) ratio of 1:4. 48 h post transfection, cells were lysed in Leeds lysis buffer for western blot [85].

### *In vitro* kinase assay

Human recombinant STAT3 (Sigma SRP2062) was incubated with 10 mU active JNK1, ERK2 or p38α in 50 mM Tris-HCl pH 7.5, 0.1 mM EGTA, 10 mM magnesium acetate, 0.1 mM Na_3_VO_4_, 0.1% (v/v) 2-mercaptoethanol and 0.1 mM [γ^32^P] ATP for 30 minutes at 30°C. The reaction was terminated by the addition of 5x SDS sample buffer (0.24 M Tris-HCl pH6.8, 8% (w/v) SDS, 40% (v/v) glycerol, 5% (v/v) 2-mercaptoethanol). Incorporation of phosphate was determined following SDS-PAGE and autoradiography or immunoblotting with pS727 STAT3 antibody. For autoradiography, the gel was stained with Instant Blue (Expedeon) for 1h at room temperature, destained in water and exposed to film at -80°C. Protein bands were excised from the gel and γ^32^P measured by Cerenkov counting in a liquid scintillation counter. The pSTAT3 western blot and the Coomassie stained gel were imaged using a LiCor Odyssey and quantified using Image Studio software.

### Western blotting

Total protein was resolved by SDS-PAGE (10–15% Tris-Glycine), transferred onto Hybond nitrocellulose membrane (Amersham biosciences) and probed with antibodies specific for Phospho-STAT3 (S727) (ab32143, abcam), Phospho-STAT3 (Y705) (9131, Cell Signalling Technology (CST)), Total STAT3 (C-20: sc-482, Santa Cruz Biotechnology), Phospho-STAT5 (Y694) (9314, CST), Phospho-JAK2 (Y1007/1008) (3776, CST), Total JAK2 (3230, CST), involucrin (SY5, Santa Cruz Biotechnology), HPV18 E6 (G-7, Santa Cruz Biotechnology), HPV18 E7 (8E2, Abcam (ab100953), HPV 16/18 E6 (C1P5, Santa Cruz Biotechnology), HPV 16 E7 (ED17, Santa Cruz Biotechnology), Phospho-ERK1/2 (Thr202/Tyr204) (43705, CST), Phospho-JNK (Thr183/Tyr185) 4668, CST), Phospho-p38 (Thr180/Tyr182) (9211, CST), Bcl xL (H-62, Santa Cruz Biotechnology), Cyclin D1 (A-12, Santa Cruz Biotechnology) p53 (FL-393, Santa Cruz Biotechnology), p21 (2947, CST), FLAG (F3165, Sigma), GFP (B-2: sc-9996, Santa Cruz Biotechnology) and GAPDH (G-9, Santa Cruz Biotechnology). Western blots were visualized with species-specific HRP conjugated secondary antibodies (Sigma) and ECL (Thermo/Pierce).

### Flow cytometry

Keratinocytes were incubated for 48 hours with STAT3 inhibitors or transfected with STAT3 siRNA, harvested and fixed in 70% ethanol overnight. The ethanol was removed and cells washed with PBS containing 0.5% BSA. Cells were stained with PBS containing 0.5% BSA, 50 μg/ml propidium iodide (Sigma) and 5 μg/ml RNase (Sigma) and incubated in this solution for 30 minutes at room temperature. Samples were processed on an LSRFortessa cell analyzer (BD) and the PI histograms analyzed on modifit software.

### Lentivirus transduction

Lentivirus plasmids were transfected into HEK 293TT cells with envelope and GAG/polymerase plasmids (kindly provided by Professor Greg Towers, University College London) using PEI transfection reagent. After 48 hours the medium was removed from the HEK 293TT cells and added to keratinocytes for 3 hours. After this time, the complete E medium was replaced and the cells incubated for 48 hours.

### Quantitative real-time PCR

Total RNA was extracted from NHK using the E.Z.N.A. Total RNA Kit I (Omega Bio- Tek) according to the manufacture’s protocol. One μg of total RNA was DNase treated following the RQ1 RNase-Free DNase protocol (Promega) and then reverse transcribed with a mixture of random primers and oligo(dT) primers using the qScript cDNA SuperMix (Quanta Biosciences) according to instructions. qRT- PCR was performed using the QuantiFast SYBR Green PCR kit (Qiagen). The PCR reaction was conducted on a Corbett Rotor-Gene 6000 (Qiagen) as follows: initial activation step for 10 min at 95°C and a three-step cycle of denaturation (10 sec at 95°C), annealing (15 sec at 60°C) and extension (20 sec at 72°C) which was repeated 40 times and concluded by melting curve analysis. The data obtained was analysed according to the ΔΔCt method using the Rotor-Gene 6000 software [86]. Specific primers were used for each gene analysed and are shown in S1 Table. U6 served as normaliser gene.

### Chromatin immunoprecipitation

ChIP experiments were carried out in primary foreskin keratinocytes containing episomal HPV18 genomes as previously described [60]. ChIP efficiency throughout the HPV18 genome was assessed by quantitative PCR (qPCR) using SensiMix SYBR master mix (Bioline). Primer sequences are available on request. STAT3 association with the cyclin D1 promoter was assessed in each individual ChIP experiment using previously described primer sequences [87].

### Statistical analysis

Where indicated, data was analyzed using a two-tailed, unpaired Student’s t-test.

## Supporting information

S1 Fig Depletion of E6 in HPV containing keratinocytes reduces STAT3 phosphorylation. **A)** Representative western blots of HPV18-containing keratinocytes transfected with a pool of two HPV18 E6 specific siRNA and analysed with antibodies specific for phosphorylated and total STAT3, HPV18 E6 and E7 and p53. GAPDH expression was used as a loading control. **B)** Representative western blots of HPV18-containing keratinocytes transfected with a HPV18 E7 specific siRNA and analysed with antibodies specific for phosphorylated and total STAT3, HPV18 E6 and E7. GAPDH expression was used as a loading control. **C**) Representative western blots of HPV18-containing keratinocytes and HPV18 E5KO-containing keratinocytes subjected to high calcium differentiation and analysed for phosphorylated and total STAT3. GAPDH serves as a loading control. **D)** Representative sections of organotypic raft cultures from HPV18 wild type and HPV18 E5KO-containing keratinocytes stained with antibodies specific for pS727 STAT3 and counterstained with DAPI to highlight the nuclei (blue in the merged panels). Images were acquired using identical exposure times. Scale bar, 20 μm. White dotted lines indicate the basal cell layer.
(TIFF)

S2 Fig Modulation of STAT3 in primary keratinocytes does not affect STAT5 phosphorylation. **A)** Representative western blot of HPV18-containing keratinocytes differentiated in high calcium media for 48 h and untreated or treated with 10 μM cryptotanshinone analysed with an antibody specific for phosphorylated STAT5. **B)** Representative western blot of HPV18-containing keratinocytes treated with 4 individual STAT3 specific siRNAs or a scramble control and analysed with an antibody specific for phosphorylated STAT5. **C)** Representative western blot of HPV18-containing keratinocytes transduced with a lentivirus encoding a STAT3 Y705F mutant or transiently transfected with a STAT3 S727A expression plasmid and analysed with an antibody specific for phosphorylated STAT5. GAPDH expression was used as a loading control in all western blots. All experiments were performed independently at least three times.
(TIFF)

S3 Fig Phosphorylation of STAT3 S727 by recombinant MAPK proteins. Recombinant STAT3 was incubated in *in vitro* kinase reactions with recombinant MSK1, JNK1, ERK2 and p38α as described in materials and methods. Proteins were analysed by SDS PAGE and protein bands excised from the gel and γ^32^P measured by Cerenkov counting in a liquid scintillation counter. Data are represented relative to a no kinase control.
(TIFF)

S4 Fig Cryptotanshinone does not cause cytotoxicity in HPV18-containing primary keratinocytes. **A)** HPV18-containing primary keratinocytes treated with increasing doses of cryptotanshinone and analyzed for cell viability by MTT assay. Bars represent the means ± standard deviation of at least three independent experiments.
(TIFF)

S5 Fig Additional images of organotypic raft cultures. **A)** Representative images of H&E stained organotypic raft cultures of NHK and HPV18-containing keratinocytes transduced with empty lentivirus or lentivirus expressing Y705F STAT3 and imaged at 40x magnification. Organotypic raft cultures of NHKs were stained with antibodies specific for **B)** cyclin B1 and **C**) involucrin. Nuclei are visualised with DAPI (blue) and white dotted lines indicate the basal cell layer. **D)** Representative sections from HPV18-containing raft cultures transduced with empty lentivirus or lentivirus expressing Y705F STAT3 and stained with an antibody specific for E1^E4. DAPI stained nuclei (Blue) and dotted white lines indicate basal layer. Widefield image 40x magnification.
(TIFF)

S1 Table A list of primer sequences used in the quantitative RT-PCR experiments. The table includes gene name and sequences of forward and reverse primers.
(TIFF)
