# Supplementary figures and images for: STAT3 activation by E6 is essential for the differentiation-dependent HPV18 life cycle
Source: PLoS Pathog. 2018 Apr 9;14(4):e1006975. doi: 10.1371/journal.ppat.1006975 (PMC5908086; doi:10.1371/journal.ppat.1006975)

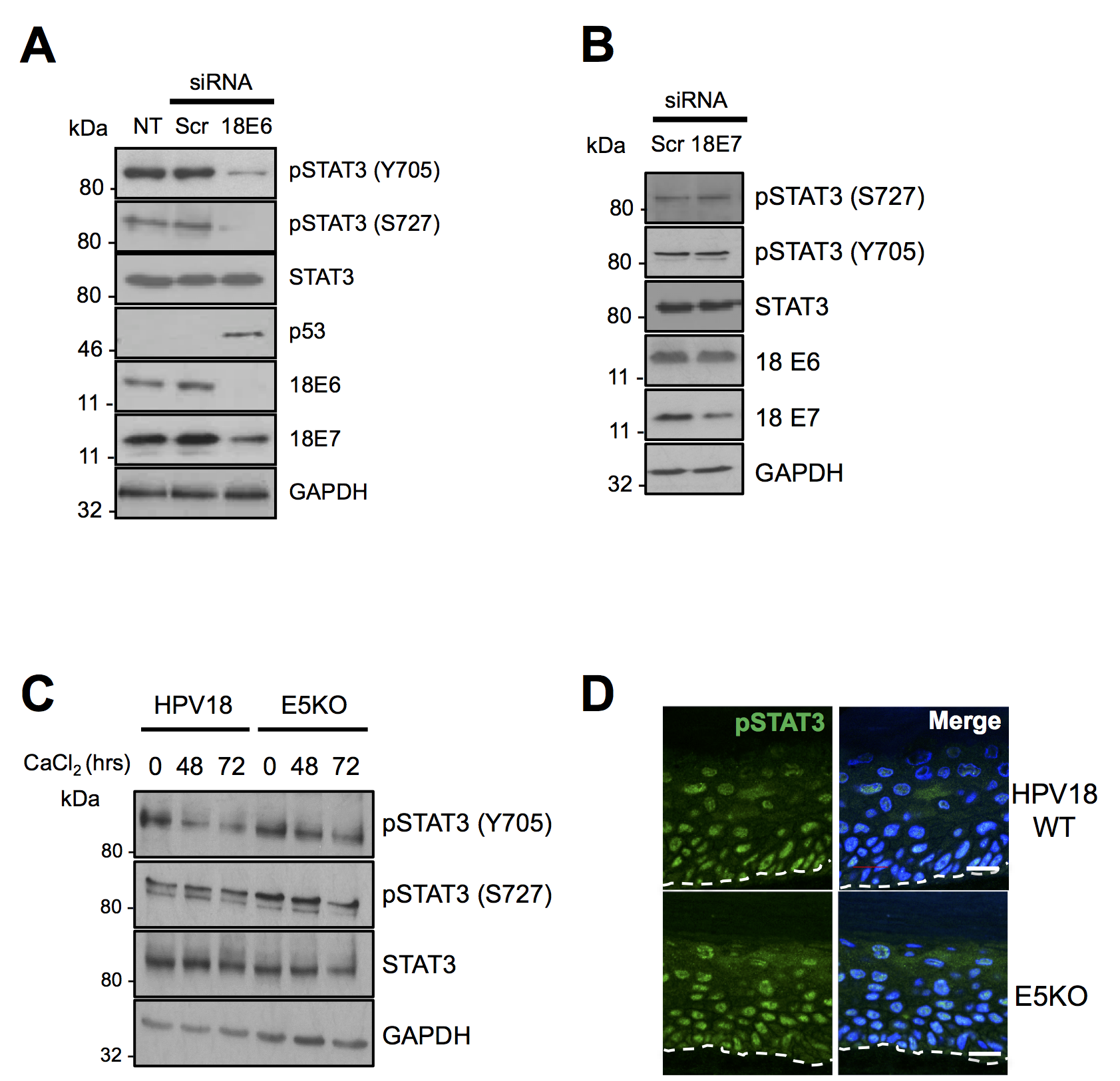

Supplement: S1 Fig — A) Representative western blots of HPV18-containing keratinocytes transfected with a pool of two HPV18 E6 specific siRNA and analysed with antibodies specific for phosphorylated and total STAT3, HPV18 E6 and E7 and p53. GAPDH expression was used as a loading control. B) Representative western blots of HPV18-containing keratinocytes transfected with a HPV18 E7 specific siRNA and analysed with antibodies specific for phosphorylated and total STAT3, HPV18 E6 and E7. GAPDH expression was used as a loading control. C) Representative western blots of HPV18-containing keratinocytes and HPV18 E5KO-containing keratinocytes subjected to high calcium differentiation and analysed for phosphorylated and total STAT3. GAPDH serves as a loading control. D) Representative sections of organotypic raft cultures from HPV18 wild type and HPV18 E5KO-containing keratinocytes stained with antibodies specific for pS727 STAT3 and counterstained with DAPI to highlight the nuclei (blue in the merged panels). Images were acquired using identical exposure times. Scale bar, 20 μm. White dotted lines indicate the basal cell layer. (TIFF) [file ppat.1006975.s001.tiff]

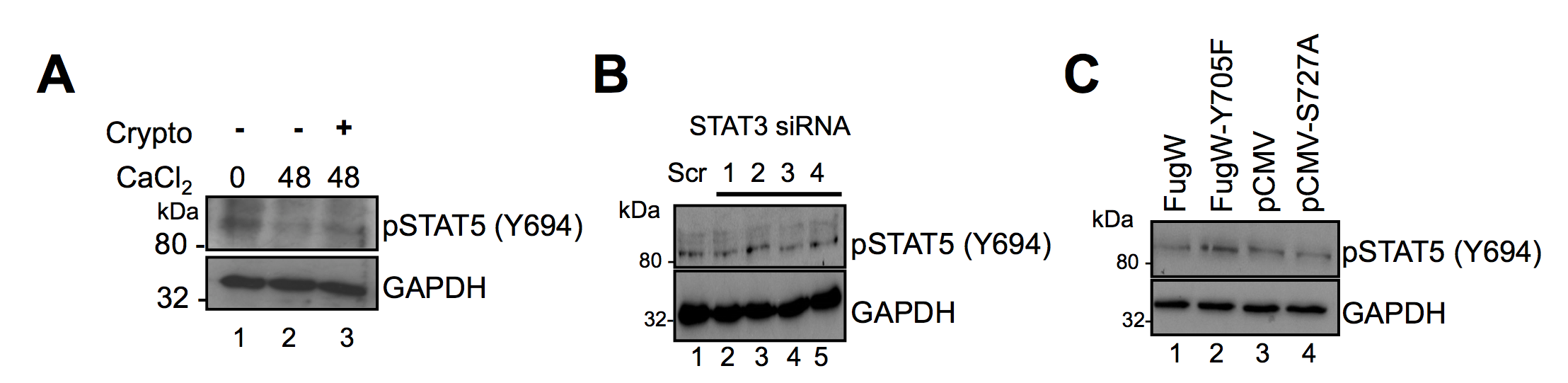

Supplement: S2 Fig — A) Representative western blot of HPV18-containing keratinocytes differentiated in high calcium media for 48 h and untreated or treated with 10 μM cryptotanshinone analysed with an antibody specific for phosphorylated STAT5. B) Representative western blot of HPV18-containing keratinocytes treated with 4 individual STAT3 specific siRNAs or a scramble control and analysed with an antibody specific for phosphorylated STAT5. C) Representative western blot of HPV18-containing keratinocytes transduced with a lentivirus encoding a STAT3 Y705F mutant or transiently transfected with a STAT3 S727A expression plasmid and analysed with an antibody specific for phosphorylated STAT5. GAPDH expression was used as a loading control in all western blots. All experiments were performed independently at least three times. (TIFF) [file ppat.1006975.s002.tiff]

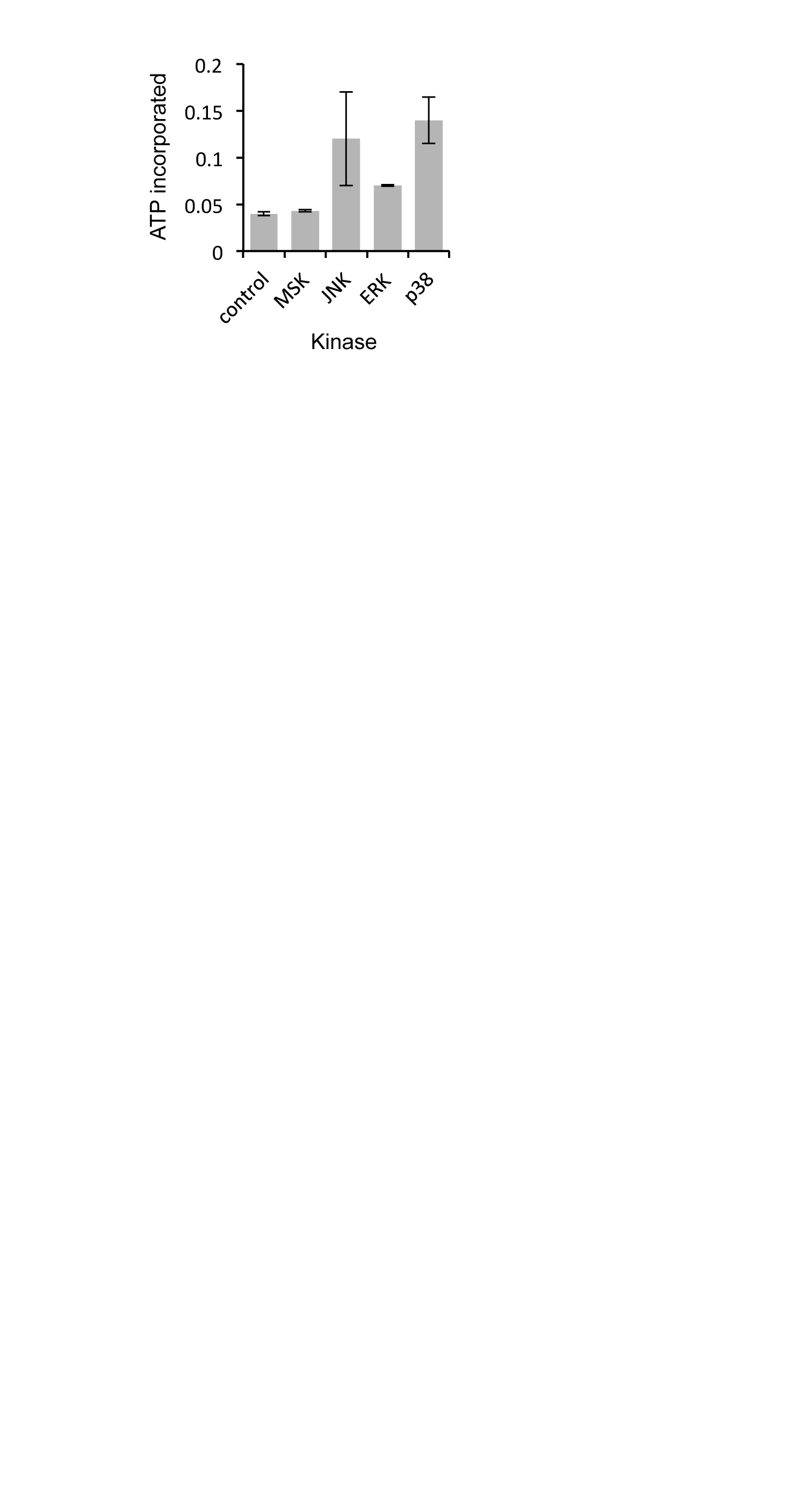

Supplement: S3 Fig — Recombinant STAT3 was incubated in in vitro kinase reactions with recombinant MSK1, JNK1, ERK2 and p38α as described in materials and methods. Proteins were analysed by SDS PAGE and protein bands excised from the gel and γ32P measured by Cerenkov counting in a liquid scintillation counter. Data are represented relative to a no kinase control. (TIFF) [file ppat.1006975.s003.tiff]

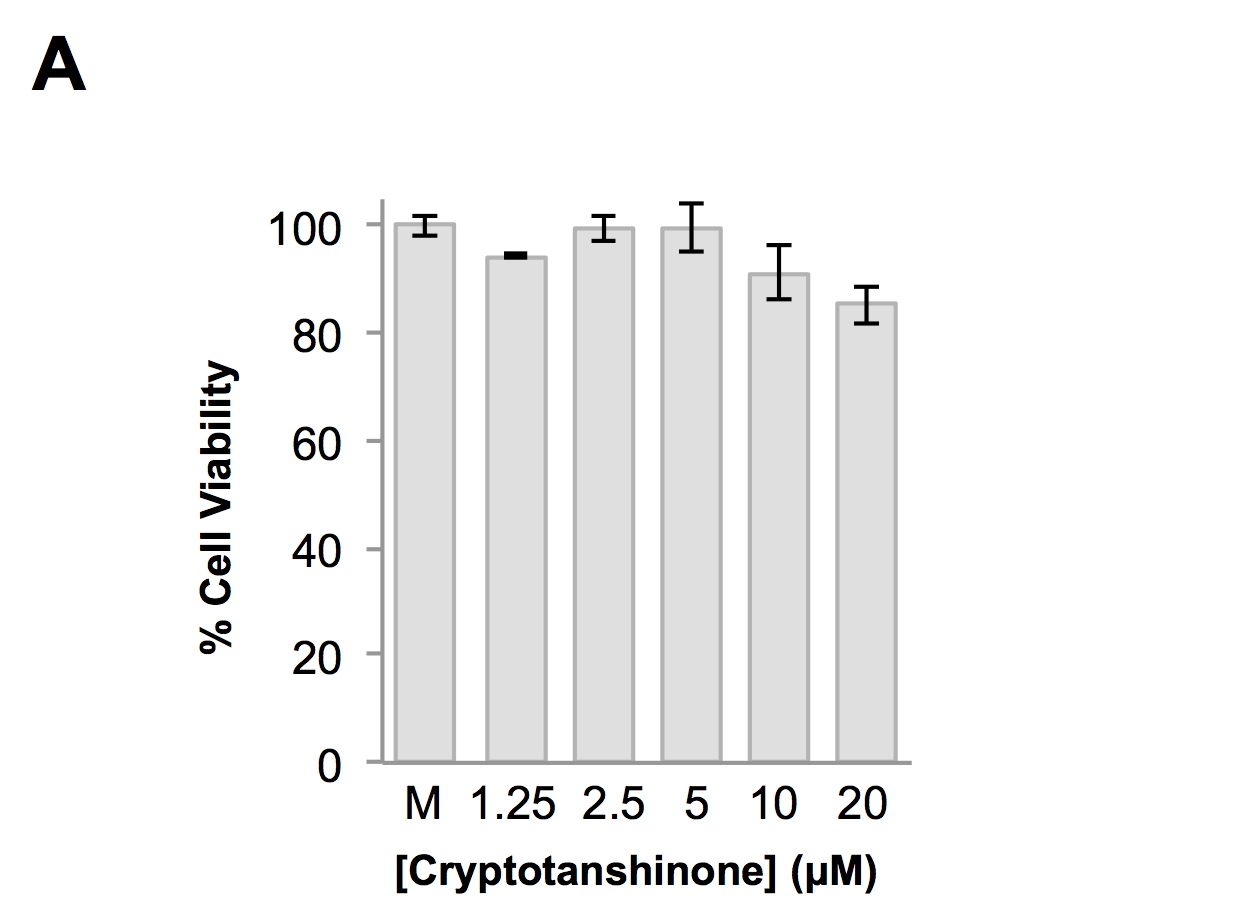

Supplement: S4 Fig — A) HPV18-containing primary keratinocytes treated with increasing doses of cryptotanshinone and analyzed for cell viability by MTT assay. Bars represent the means ± standard deviation of at least three independent experiments. (TIFF) [file ppat.1006975.s004.tiff]

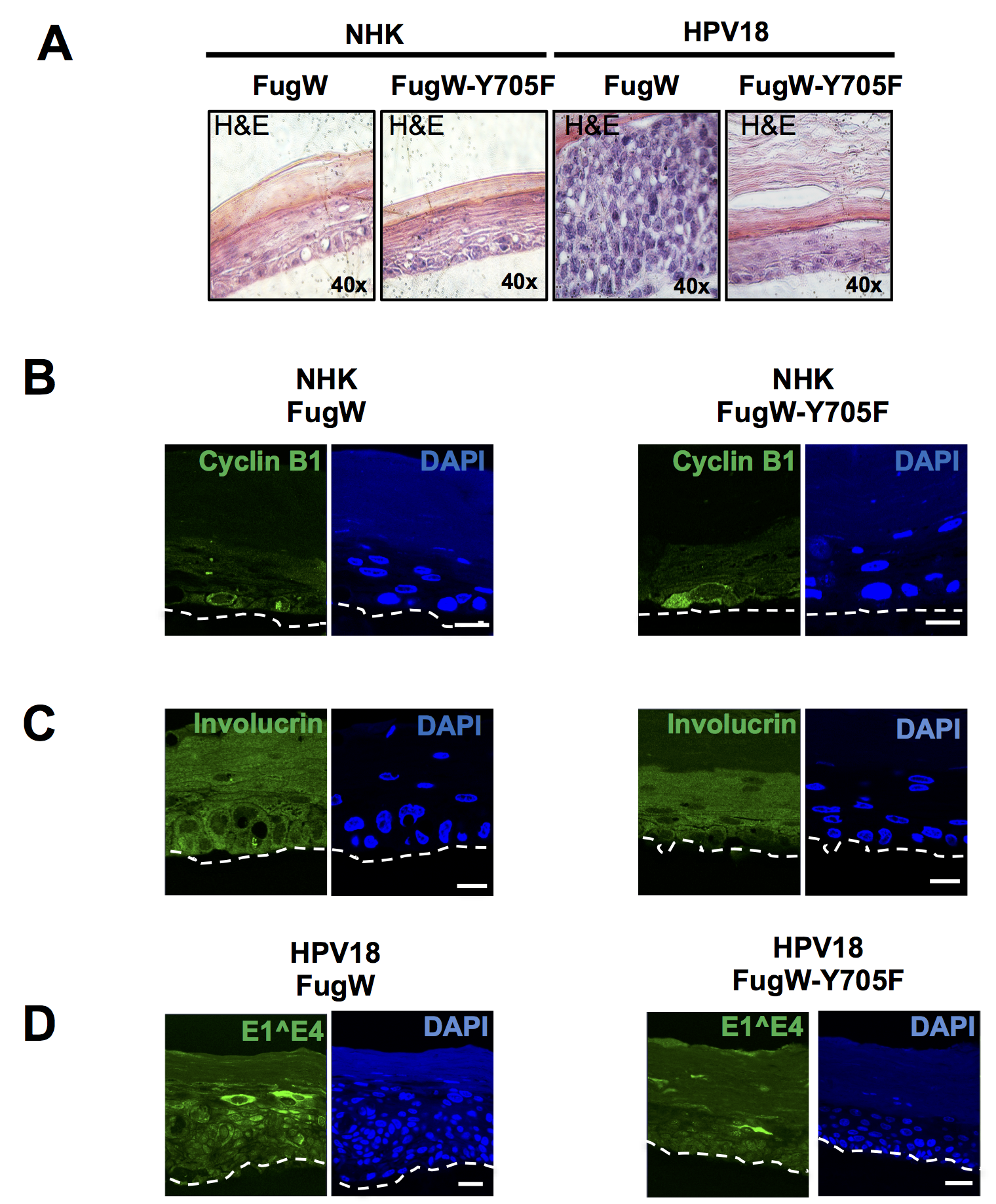

Supplement: S5 Fig — A) Representative images of H&E stained organotypic raft cultures of NHK and HPV18-containing keratinocytes transduced with empty lentivirus or lentivirus expressing Y705F STAT3 and imaged at 40x magnification. Organotypic raft cultures of NHKs were stained with antibodies specific for B) cyclin B1 and C) involucrin. Nuclei are visualised with DAPI (blue) and white dotted lines indicate the basal cell layer. D) Representative sections from HPV18-containing raft cultures transduced with empty lentivirus or lentivirus expressing Y705F STAT3 and stained with an antibody specific for E1^E4. DAPI stained nuclei (Blue) and dotted white lines indicate basal layer. Widefield image 40x magnification. (TIFF) [file ppat.1006975.s005.tiff]

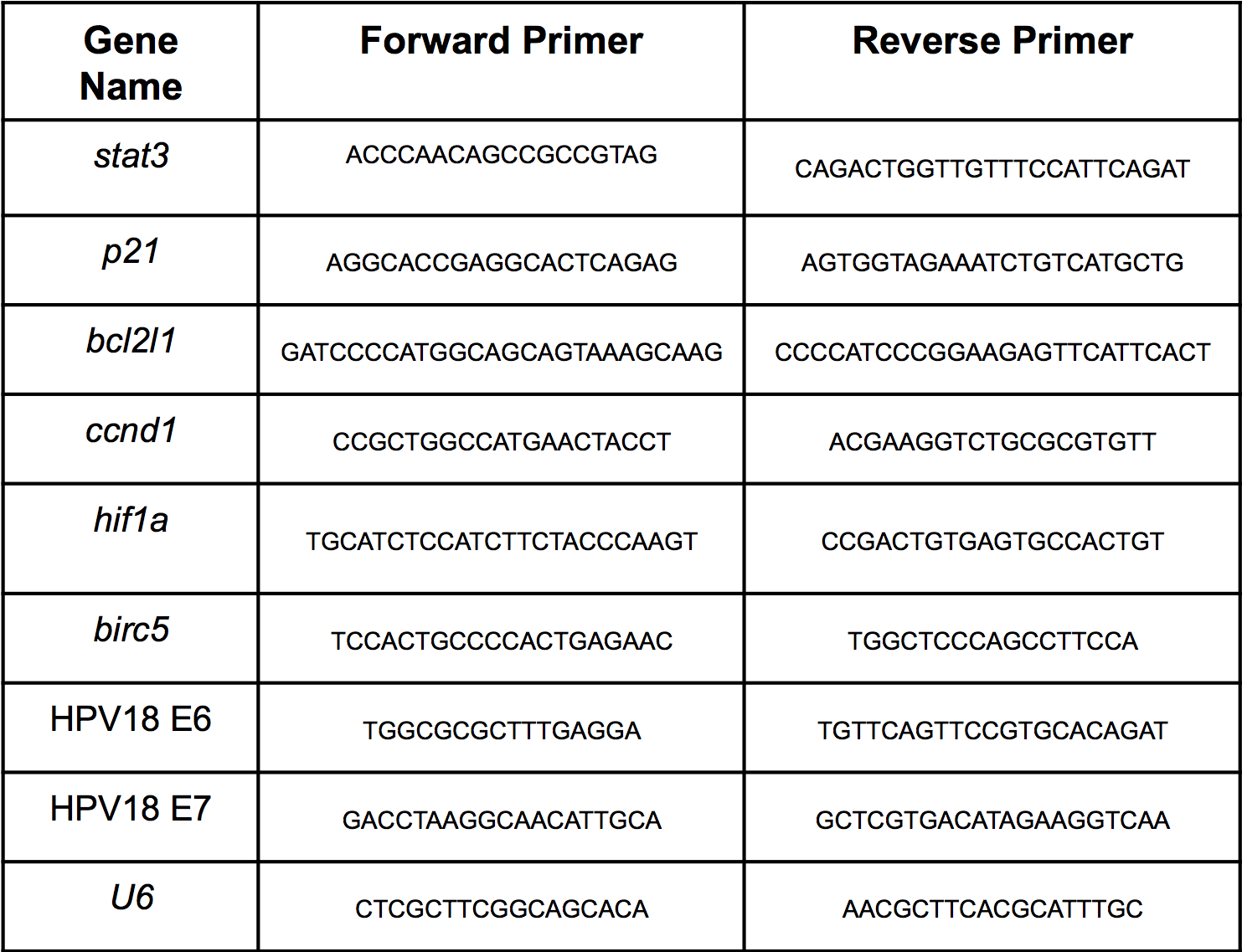

Supplement: S1 Table — The table includes gene name and sequences of forward and reverse primers. (TIFF) [file ppat.1006975.s006.tiff]
